# Signaling Mediated by Toll-*Like* Receptor 5 Sensing of *Pseudomonas aeruginosa* Flagellin Influences IL-1β and IL-18 Production by Primary Fibroblasts Derived from the Human Cornea

**DOI:** 10.3389/fcimb.2017.00130

**Published:** 2017-04-19

**Authors:** Maria del Mar Cendra, Myron Christodoulides, Parwez Hossain

**Affiliations:** ^1^Division of Clinical and Experimental Sciences, Department of Molecular Microbiology, Faculty of Medicine, University of SouthamptonSouthampton, UK; ^2^Eye Unit, Division of Clinical and Experimental Sciences, Faculty of Medicine, University of SouthamptonSouthampton, UK

**Keywords:** *Pseudomonas aeruginosa*, fibroblasts, human cornea, cytokine, NLRC4, flagellin, MyD88, TLR5

## Abstract

*Pseudomonas aeruginosa* is the principal cause of bacterial keratitis worldwide and overstimulation of the innate immune system by this organism is believed to contribute significantly to sight loss. In the current study, we have used primary human corneal fibroblast (hCF) cells as an *ex vivo* model of corneal infection to examine the role of *P. aeruginosa* flagellum and type three secretion system (TTSS) in inducing inflammasome-associated molecules that trigger IL-1β and IL-18 production during the early stages of the infection. Our results show that *P. aeruginosa* infection stimulated the non-canonical pathway for IL-1β and IL-18 expression and pathway stimulation was influenced predominantly by the flagellum. Both IL-1β and IL-18 cytokines were expressed intracellularly during bacterial infection, but only the former was released and detected in the extracellular environment. We also investigated the signaling pathways in hCFs mediated by Toll-*Like* Receptor (TLR)4 and TLR5 sensing of *P. aeruginosa*, and our data show that the signal triggered by TLR5-flagellin sensing significantly contributed to IL-1β and IL-18 cytokine production in our model. Our study suggests that IL-18 expression is wholly dependent on extracellular flagellin sensing by TLR5, whereas IL-1β expression is also influenced by *P. aeruginosa* lipopolysacharide. Additionally, we demonstrate that IL-1β and IL-18 production by hCFs can be triggered by both MyD88-dependent and -independent pathways. Overall, our study provides a rationale for the development of targeted therapies, by proposing an inhibition of flagellin-PRR-signaling interactions, in order to ameliorate the inflammatory response characteristic of *P. aeruginosa* keratitis.

## Introduction

Bacterial keratitis is a global health problem in which pathogen-induced cytotoxicity and overstimulation of the host innate inflammatory response can lead to severe visual impairment and sight lost (Taube et al., 2015). In the human eye, superficial damage and surface laceration are often associated with contact lens wear. Thus, when the corneal epithelium barrier and Bowman's membrane located between the epithelium and the stroma in the cornea are physically breached, microbes can penetrate and establish infection in the stroma. Keratitis is characterized by the production of inflammatory cytokines, the subsequent influx of polymorphonuclear leukocytes (PMNL) and extensive tissue damage. As a consequence of microbial penetration into the stroma, resident keratocytes can respond by transforming into corneal fibroblasts that exacerbate the inflammatory response (O'brien, 2003). *Pseudomonas aeruginosa* is the leading cause of bacterial keratitis associated with contact lens wear (Shah et al., 2011; Stapleton and Carnt, 2012).

Stimulation of the host innate immune response is initiated by pattern recognition receptors (PPR), which are immune receptors that recognize pathogen-associated molecular patterns (PAMPs) of infectious agents and trigger different types of cellular responses. Expression of cell wall lipopolysaccharide (LPS) O-antigen, the exotoxin injector type three secretion system (TTSS) and flagellum are key *P. aeruginosa* PAMPs (Hauser, 2011; Le Berre et al., 2011). The TTSS is a molecular machine encoded by pathogenic gram-negative bacteria, which is used to meditate interactions with eukaryotic host cells. This bacterial complex consists of a syringe-like “injectisome” that enables active translocation of bacterial proteins into the host cell cytoplasm, in order to alter eukaryotic cell biology during infection (Notti and Stebbins, 2016). Intracellular delivery of exotoxins is sensed by the host immune system: intracellular PPRs sense conserved bacterial determinants that have been internalized, e.g., components of the injectisome, thus activating a host response to infection (Puhar and Sansonetti, 2014). For *P. aeruginosa*, the PopB, PopD and PcrV are essential TTSS proteins for the exotoxin delivery mechanism. Possession of the TTSS has been suggested to be indispensable for PAO1 strain in promoting tissue damage and keratitis in animal models (Pier, 2007; Hauser, 2009). *P. aeruginosa* flagellin (FliC) is the protein product of the *fliC* gene, which becomes the flagellum filament when it is polymerized (Hayashi et al., 2001; Berg, 2003; Ghosh, 2004). Toll-*like* receptors (TLRs) are a family of PPRs and classified depending on whether they are found anchored within the cell membrane or located intracellularly (Janeway and Medzhitov, 2002; Kawasaki and Kawai, 2014). In the current study, we have focused on investigating cell membrane-associated TLR2, which senses bacterial lipoproteins; TLR4, which is activated by LPS, a classical inducer of host inflammatory processes; and TLR5, which is activated by extracellular flagellin that can subsequently be delivered within the cytosol (Kawasaki and Kawai, 2014). When stimulated, TLRs recruit TIR^1^ domain-containing adaptors, such as MyD88^2^, TRIF^3^, TIRAP^4^/MAL^5^, or TRAM^6^, and activate subsequent signaling pathways that lead to transcription of NF-κβ^7^ and IRFs^8^ to orchestrate a wide range of inflammatory responses and cytokine expression (O'neill et al., 2013; Kawasaki and Kawai, 2014). *P. aeruginosa* has been reported to activate TRIF and MyD88 adaptor molecules in mouse macrophages, but not TIRAP (Sun et al., 2010).

Nod-*like* receptors (NLRs) are another family of PPRs, but they are found within the cytosol. NLRs sense intracellular PAMPS and promote the assembly of a multi-protein scaffold called the “inflammasome” (Chen et al., 2009; O'neill et al., 2013). The inflammasome recruits and activates certain caspases for maturing interleukin (IL)-1β and IL-18 cytokine pro-forms, and also induces a cell-death program called “pyroptosis” (Schroder and Tschopp, 2010; Latz et al., 2013). To date, numerous inflammasome-associated proteins have been described including NLRP^9^1, NLRP2, NLRP3, AIM2^10^, and NLRC^11^4 (Schroder and Tschopp, 2010; Ozaki et al., 2015). *P. aeruginosa* flagellin and TTSS have been reported to be important for the intracellular assembly of the non-canonical inflammasome NLRC4 in mouse models (Sutterwala et al., 2007; Miao et al., 2010; Zhao et al., 2011).

It is generally accepted that PAMP recognition by host cell PRR leads to the production of cytokines as well as other innate immune mediator signals (Mogensen, 2009). Herein, we have studied the role of the flagellum, TTSS and LPS PAMPs in the activation of inflammasome-associated signaling molecules, and consequential IL-1β and IL-18 production by human primary stromal corneal fibroblasts (hCFs), during the early stages of *P. aeruginosa* infection. The rationale for studying IL-1β and IL-18 expression in our *in vitro* model of hCFs is based on our observation that infection of these cells with wild-type *P. aeruginosa* is characterized predominantly by the production of these two cytokines, with the expression of other cytokines, chemokines, and immune mediator molecules not significant (manuscript submitted elsewhere). Furthermore, we have tested the hypothesis that inhibition of intracellular signaling pathways during *P. aeruginosa* infection can lead to reduced pro-inflammatory cytokine expression in hCFs.

## Materials and methods

### Human primary stromal corneal fibroblast (hCF)

Corneal buttons were collected aseptically from patients at the eye unit of a large hospital in the south of England. After removing the corneal epithelium, the stromal layer was dissected and digested with collagenase Type-1 (1 mg/mL; Life Technologies, Warrington, UK) for 3 h at 37°C. Digested stroma was cultured in Corneal Culture Medium (CCM) and hCF were characterized as described previously (Wong et al., 2011). Human monocytic cell line THP-1 was cultured in RPMI 1640 containing glutamine (Lonza, Biologics, Tewkesbury, UK), 1% (v/v) Penicillin-Streptomycin and 10% (v/v) decomplemented Fetal Calf Serum (dFCS; Life Technologies, Warrington, UK). Cells were cultured in a humidified incubator at 37°C and 5% (v/v) CO_2_.

### Ethics statement

Patients provided written informed consent to use surplus corneal tissue specimen for research via the NHS Blood Transplant Eye Retrieval Service based at Southampton General Hospital (Southampton, UK). Protocols were approved by the NRES Committee South Central-Berkshire 06/Q1602/56.

### Bacterial strains, plasmids, and growth conditions

*Pseudomonas aeruginosa* strain PAO1 (Holloway1C Stanier131) was obtained from the National Collection of Industrial, Food and Marine Bacteria, UK. *P. aeruginosa* strain PA14 and PA14 Δ*flgK* and PA14 Δ*popB* mutants were provided by George O'Toole (Dartmouth Medical School, Dartmouth, USA). PA14 Δ*flgK* mutant is unable to synthesize a functional flagellum and the PA14 Δ*popB* mutant lacks the PopB protein, an essential protein without which the TTSS cannot deliver exotoxins within eukaryotic cells (Berg, 2003; Hauser, 2009). Green Fluorescence Protein (GFP) was inserted into PAO1, PA14 and mutant strains by transformation of the PAO1/GFP plasmid provided by Alice Prince (Columbia University, New York, USA) (Parker and Prince, 2013). To complement PA14 Δ*flgK* and PA14 Δ*popB* mutation, the *flgK* and *popB* coding region sequences from the PA14 genome were amplified using the primers listed in Supplementary Table 1 and cloned into the HindIII and BamHI sites of the shuttle cloning vector pUCP19 (Schweizer, 1991). pUCP19-*flgK* and pUCP19-*popB* were transformed into *Escherichia coli* DH5α and into PA14 Δ*flgK* and PA14 Δ*popB* strains (Irani and Rowe, 1997). Carbenicillin (Sigma-Aldrich, Poole, UK) at 100 and 300 μg/mL was added to the medium for plasmid maintenance for *E. coli* and *P. aeruginosa*, respectively. Bacterial strains were grown in Luria-Bertani broth and agar (Oxoid, Basingstoke, UK) at 37°C.

### Transfection of hCFs with TLR5-siRNA (siTLR5-hCFs)

hCFs were grown to 30–50% of confluence in a 24-well plate and transfected with four TLR5 siRNA (FlexiTube GeneSolution, Qiagen, Manchester, UK) using INTERFERin® (Polyplus transfection, Illkirch-Graffenstaden, France). Mock RNA was used as a transfection control. A total of 1 nM of TLR5-siRNA per well was mixed with 100 μl of dFCS-free CCM and 2 μl of INTERFERin®. After 10 min at room temperature (RT), the transfection mix was added to the CCM (500 μl) already present in the wells and incubated for 4 h. Transfection solution was then removed and 1 mL of fresh CCM was added per monolayer and cells were incubated for 48 h. Mock transfection with an irrelevant siRNA was done to ensure that TLR5 expression remained unaltered. TLR5 silencing was checked by measuring TLR5 expression in PA14-stimulated hCFs by Real Time quantitative Polymerase Chain Reaction (RT-qPCR) and western-blot.

### Expression and purification of recombinant (r) FliC protein of *P. aeruginosa*

The *fliC* (flagellin) coding region of PAO1 is 100% identical to that in PA14, and it was amplified using the primers listed in Supplementary Table 1 and cloned into the NdeI and XhoI restriction sites of the T7 promoter vector pET22b(+) (Merck Millipore, Hertfordshire, UK). After checking pET22b-*fliC* by PCR with T7 universal oligonucleotides (Supplementary Table 1) and sequencing, the plasmid was transformed into *E. coli* BL21 using ampicillin (Sigma-Aldrich, Poole, UK) at 50 μg/mL to maintain the plasmid. BL21 pET22b-*fliC* culture was grown to an OD λ_600_ nm = 0.5 and induced with isopropyl-β-D-1-thiogalactopyranoside (IPTG; 1 mM) for 4 h, harvested and lysed. Cell supernatant and pellet from the lysed culture were analyzed by SDS-PAGE, which demonstrated presence of the recombinant (r)FliC in the supernatant. rFliC was purified from the soluble fraction of harvested cultures using nickel-nitrilotriacetic acid (Ni-NTA) metal-affinity chromatography (Qiagen, Manchester, UK) under native conditions, as described previously (Hung et al., 2015). Bound rFliC was eluted with 50 mM NaH_2_PO_4_, 300 mM NaCl, 250 mM imidazole buffer (pH 8.0) and elution fractions analyzed by SDS-PAGE. Pooled fractions were dialyzed against PBS, pH7.4 for 2 days. Lipopolysaccharide (LPS) content was quantified with the Pierce LAL Chromogenic Endotoxin Quantitation Kit (Thermo Fisher Scientific, Basingstoke, UK) following the manufacturer's instructions. SDS-PAGE of purified rFliC confirmed the purity and molecular mass (~49 KDa) of the protein (Supplementary Figure 1).

### Infection of hCFs with *P. aeruginosa* strains and different stimulatory conditions

hCFs were seeded into 24-well culture plates (Greiner bio-one, Stonehouse, UK, ~10^5^ cells/well) and infected over time with *P. aeruginosa* at a Multiplicity Of Infection (MOI) of 10 in dFCS-free DMEM medium (Sigma-Aldrich, Poole, UK). Stimulatory conditions included 100 ng/mL of *P. aeruginosa* LPS (Sigma-Aldrich, Poole, UK) and 5 μg/mL of purified *P. aeruginosa* rFliC protein for 6 h and when required, streptolysin-O (SLO), was used for the intracellular delivery of the molecule as described previously (Walev et al., 2001). Bacterial association to hCFs was quantified over time as described previously (Hardy et al., 2000). Briefly, monolayers were washed gently 4 times with phosphate buffered saline (PBS), pH7.4 and lysed with 250 μl of PBS containing saponin [1% (w/v); Sigma-Aldrich, Poole, UK] and dFCS (1% w/v) for 15 min. Determination of colony forming units was done by serial dilution and viable counting on agar plates.

### Signaling pathway inhibition

Inhibition experiments were done using 50 μM of Myd88 peptide inhibitor (MyD88i, Bio-techne, Abington, UK) and 1 μM of TAK-242 (Merck Millipore), respectively (Loiarro et al., 2005; Takashima et al., 2009; Matsunaga et al., 2011). hCFs were treated with MyD88i and TAK-242 for 24 and 12 h, respectively, prior to hCFs stimulation with *P. aeruginosa* strains, rFliC or LPS for 6 h. Uninfected hCFs monolayers in inhibited and non-inhibited conditions were used as controls.

### Total RNA extraction and real time quantitative polymerase chain reaction (RT-qPCR)

In this study, total RNA was extracted from infected- and stimulated- hCF cells during different experimental conditions including hCF infection with the PAO1, PA14 wild-type and Δ*flgK*, Δ*popB* mutant strains for 1, 3 or 6 h, and after stimulation with rFliC (5 μg/mL) and *P. aeruginosa* LPS (100 ng/mL) for 6 h. Total RNA was extracted from siTLR5-hCFs monolayers after 6 h of similar stimulatory and infection conditions. RNA extraction was done using the RNeasy Mini Kit (Qiagen, Manchester, UK). Control conditions were uninfected and un-stimulated hCFs or siTLR5-hCFs. RNA samples were treated with DNAse-50 (PrimerDesign, Chandler's Ford, UK) and total concentration was quantified using a NanoDrop spectrophotometer (ND-1000, NanoDrop, Thermo Fisher Scientific, Basingstoke, UK). cDNA was synthetized from 1 μg of RNA using the RT-nanoscript system (PrimerDesign, Chandler's Ford, UK) and the reverse oligonucleotides listed in Supplementary Table 1. RT-qPCRs were done with 1 μl of each cDNA in a Rotorgene-Q 5plex HRM (Qiagen, Manchester, UK) using the Power SYBR green PCR Master Mix (Life Technologies, Warrington, UK) and oligonucleotides listed in Supplementary Table 1. β-actin was used as an endogenous control and melting curves were done to ensure specific gene amplification. The 2^−ΔΔCT^ method was used for data analyses (Livak and Schmittgen, 2001).

### Protein extraction and western blot analysis

Intracellular protein analysis of TLR2, TLR4, TLR5, NLRC4, caspase-1, caspase-4, and the cytokines IL-18 and IL-1β was done after 6 h in similar infection and stimulatory conditions, including the experiments with siTLR5-hCFs cells, as for RT-qPCR experiments. hCFs infected with complemented PA14 Δ*flgK* pUCP19-*flgK* and PA14 Δ*popB* pUCP19-*popB* strains were included for protein analyses. Intracellular mature IL-18 and IL-1β were also measured in hCFs treated with TAK-242, MyD88i and TAK-242/MyD88i and infected with PA14 and mutant strains. Cells were washed in PBS and total protein extract was obtained by using Cytobuster™ protean extraction reagent (Merck Millipore, Nottingham, UK). Total protein (5 μg) was separated by sodium dodecyl sulfate polyacrylamide gel electrophoresis (SDS-PAGE) and electro-transferred onto polyvinylidene difluoride (PVDF) membranes (GE Healthcare, Amersham, UK). Membranes were blocked for 1 h in PBS containing 0.05% (v/v) Tween20 (PBST) and 5% (v/v) dry milk powder. Antibodies used were rabbit monoclonal anti-TLR2, -TLR4, -TLR5; rabbit polyclonal anti-NLRP3, -Caspase-4, -IL-18, and -IL-1β (Abcam, Bristol, UK); rabbit polyclonal anti-NLRC4 (Bio-techne Ltd, Abingdon, UK). All antibodies were used at the manufacturers' recommended dilutions and left overnight at 4°C. Membranes were washed three times in PBST and incubated for 1 h with goat anti-Rabbit IgG (H&L)-HRP pre-adsorbed secondary antibody (Abcam, Bristol, UK) according to the manufacturer's recommendations. Rabbit anti-β-actin polyclonal antibody (Abcam, Bristol, UK) was used as an endogenous and loading control. Immuno-detection was carried out in a Versadoc 4000 and data analyzed with Quantity One 4.6.9 software (BioRad, Langford, UK).

### Cytokine release analysis

Extracellular IL-18 and IL-1β cytokines were quantified with the Meso Scale Discovery (MSD) electro-chemiluminescence assays (Meso Scale Diagnostics, Gaithersburg, USA.) and by western blot after protein precipitation with trichloroacetic acid (TCA). Supernatants from triplicate hCF monolayers infected with wild-type PAO1, PA14, mutant PA14 Δ*flgK*, and PA14 Δ*popB* strains (MOI = 10) were collected after 6 h of infection.

### Confocal microscopy

For visual comparison of bacterial association with hCFs, hCF monolayers, grown on borosilicate glass cover slips, were infected for 9 h with GFP-expressing wild-type PAO1, PA14 and mutant PA14 Δ*flgK* and PA14 Δ*popB* strains (MOI = 10). After infection, cells were washed three times with warmed DMEM medium and *live-*stained with Wheat Germ Agglutinin (1/1,000) for 10 min at RT. Monolayers were washed with PBS and fixed with paraformaldehyde (4% v/v) for 15 min at RT. After three more PBS washes, cells were permeabilized with PBS containing saponin 0.1% (v/v) and 10% (v/v) dFCS for 2 h at 4°C and counterstained with PBS containing DAPI (1/1,000) for 15 min at RT. hCFs monolayers were washed three times in PBS and examined with a Leica SP5 LSCM confocal microscope (Leitz).

### Statistics

Data were analyzed by one-way ANOVA with Dunnett's multiple comparison test. *P* < 0.05 denoted significance.

## Results

### *Pseudomonas aeruginosa* flagellum influences bacterial attachment to hCFs

In preliminary experiments, hCFs were infected over time with different MOI (1-100) of *P. aeruginosa*. These experiments demonstrated that a MOI of 10 was optimal for infecting hCFs in order to study both logarithmic association of *P. aeruginosa* to hCFs and maximal gene and protein expression in intact cell monolayers (data not shown).

**Figure 1:**
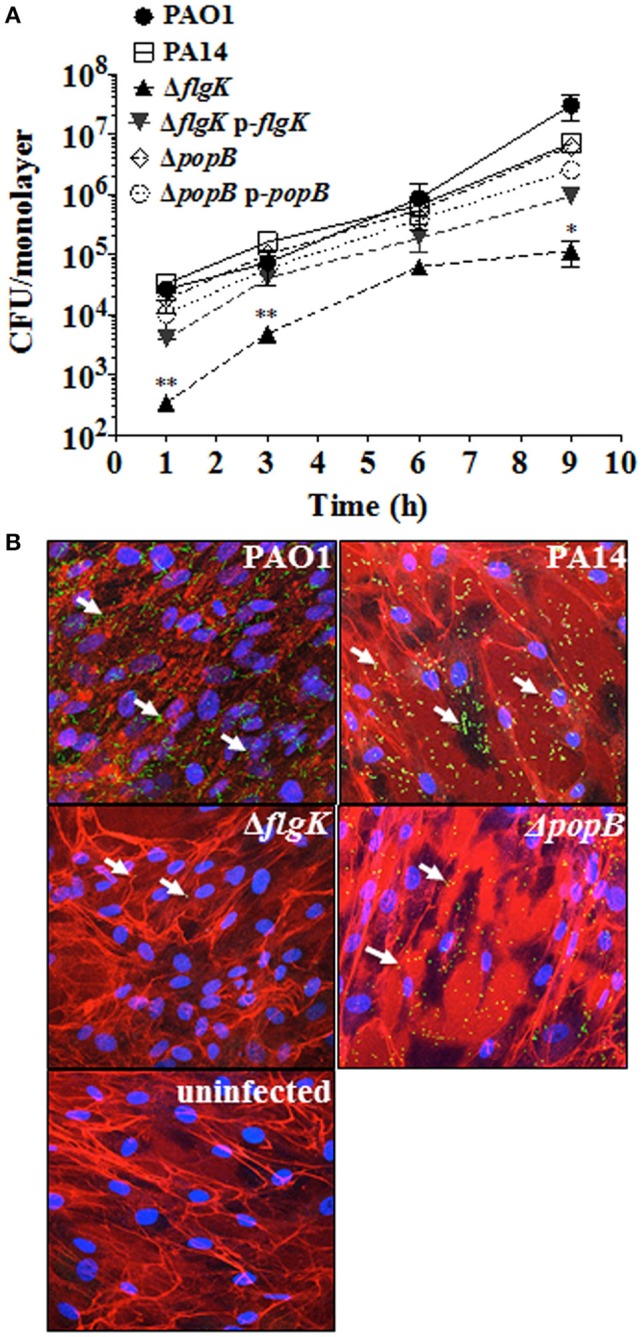
**Expression of flagellum contributes to *P. aeruginosa* adhesion to hCFs. (A)** Cells were infected with MOI = 10 of PAO1, PA14, Δ*flgK*, Δ*popB* and complemented PA14 Δ*flgK* p-*flgK*, PA14 Δ*popB* p-*popB* strains and association to hCFs monolayers was quantified by viable counting after 1, 3, 6 and 9 h. The symbols represent the means and bars the standard error of the mean (SEM) of *n* = 3 independent experiments. PA14 Δ*flgK* and PA14 Δ*popB* strains carrying the cloning vector pUCP19 without insert were tested in order to exclude possible vector interferences: no differences were observed when compared with their respective mutant strains in their association capabilities (data not shown). Statistical significance was calculated with a one-way ANOVA with Dunnett's multiple comparison test and it is represented by asterisks as follows: ^*^P < 0.05 and ^**^P < 0.01. **(B)** Confocal microscopy images of GFP-expressing PAO1, PA14 and mutant strains after 9 h of infection. Arrows denote the positions of bacteria and images are representative of *n* = 3 independent experiments.

Adhesion patterns of *P. aeruginosa* wild-type bacteria and mutant strains for flagellum (Δ*flgK*) and TTSS (Δ*popB*) to hCFs were first examined as an initial step required for cellular PRR stimulation. No significant differences (*P* > 0.05) in the association dynamics of wild-type PAO1 and PA14 strains and the PA14 Δ*popB* mutant with hCF monolayers were observed (Figure 1A). By contrast, the PA14 Δ*flgK* mutant showed a significant ~1 log reduction in bacterial association with hCFs compared to the wild-type and Δ*popB* strains at every time point examined (*P* < 0.05). The complemented PA14 Δ*flgK* pUCP19-*flgK* strain showed a similar association pattern (*P* > 0.05) as the wild-type PA14 strain by up to 6 h, but by 9 h there was an ~1 log divergence in adherence of the complemented strain compared with the parent strain (Figure 1A). There was no significant difference in adherence between the PA14 wild-type strain, the Δ*popB* mutant and the complemented PA14 Δ*popB* pUCP19-*popB* strain over the 9 h time period (*P* > 0.05; Figure 1A). Representative confocal microscopy images confirmed visually that the numbers of PA14 Δ*flgK* bacteria associated to hCFs monolayers were reduced compared to PA14 wild-type strain and Δ*popB* mutant (Figure 1B). Bacterial growth rates were similar for all the strains (Supplementary Figure 2), except for the PA14 Δ*flgK* mutant, which was marginally slower between 0 and 3 h compared with the wild-type PA14 strain, although the growth rates of both were similar thereafter (*P* > 0.05). In addition, cytotoxicity induced by *P. aeruginosa* infection (MOI = 10) as determined by release of lactate dehydrogenase enzyme was not significant by 9 h (<5% observed enzyme release, data not shown). Death of the cell monolayers occurred between 9 and 24 h of infection, regardless of phenotype (data not shown).

### *Pseudomonas aeruginosa* flagellum and TTSS enhance non-canonical inflammasome signaling molecule expression

**Figure 2:**
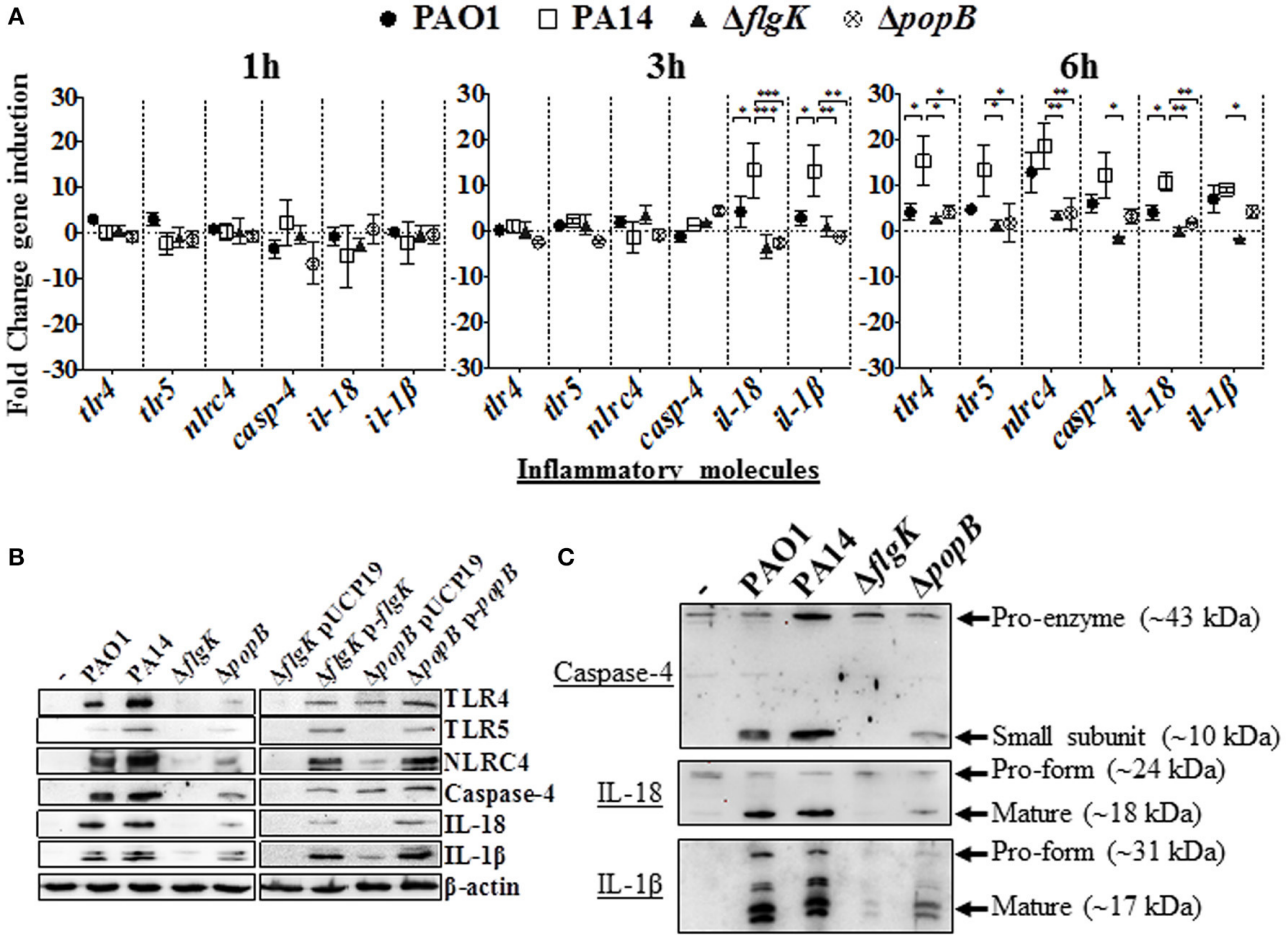
**Inflammatory responses of human corneal fibroblast cells (hCFs) toward *P. aeruginosa* infection. (A)** RT-qPCR of immune signaling molecules after 1, 3 and 6 h hCFs infection with PAO1 and PA14 wild-type and PA14 Δ*flgK* and PA14 Δ*popB* mutant strains at a MOI = 10. Symbols represent the mean and bars the SEM of the log of gene expression from infected hCFs compared with uninfected controls from *n* = 10 independent hCF samples. Validation of the oligonucleotides used for RT-qPCR examination of gene expression in infected hCFs was done by demonstrating the same signals in THP-1 cells stimulated with 100 ng/mL of *P. aeruginosa* LPS (data not shown). Statistical significance was calculated with a one-way ANOVA with Dunnett's multiple comparison test and it is represented by asterisks as follows: ^*^P < 0.05, ^**^P < 0.01, ^***^P < 0.001. **(B)** Cells infected with MOI = 10 of PAO1 and PA14 wild-type, PA14 Δ*flgK* and PA14 Δ*popB* mutant strains, and the complemented PA14 Δ*flgK* p-*flgK*, PA14 Δ*popB* p-*popB* and control PA14 Δ*flgK* pUCP19 and PA14 Δ*popB* pUCP19 strains were lysed and intracellular protein levels of TLR4, TLR5, NLRC4, active caspase-4 and matured forms of IL-18 and IL-1β were detected by western blot. Western blot image is representative of three independent experiments. **(C)** Western blot images show protein levels of pro- and matured forms of caspase-4, IL-18 and IL-1β in hCFs infected with PAO1 and PA14 wild-type and PA14 Δ*flgK* and PA14 Δ*popB* mutant strains. Protein expression for uninfected hCFs is also shown. The rabbit polyclonal anti- caspase-4, -IL-18, and -IL-1β antibodies are specific for binding to the mature form of each respective protein and with less affinity for the pro-forms. Respective molecular weights of pro and mature forms are denoted with arrows. A representative image from *n* = 5 independent experiments is shown.

Induction of inflammasome-associated signaling molecules in hCFs during the early stages of infection by *P. aeruginosa* and the effects caused by the Δ*flgK* and Δ*popB* mutations were analyzed. Expression of the *tlr2, tlr4, tlr5, nlrp3* and *nlrc4* receptor genes, *il-18* and *il-1*β cytokine genes and *caspase-1* and *caspase-4* genes was quantified by RT-qPCR after 1, 3 and 6 h of bacterial infection (Figure 2A). Each point in the graph represents the fold-change in expression caused by the wild-type and mutant bacteria compared against uninfected cells, using the 2^−ΔΔCT^ method. No significant changes in expression were observed for *tlr2, nlrp3* or *caspase-1* (data not shown). The highest levels of gene induction were observed 6 h after infection with wild-type PAO1 and PA14 strains (Figure 2A), with significant increases at 3 h observed for *il-18* and *il-1*β only with PA14 (*P* < 0.05). A one-way ANOVA with Dunnett's test was used to compare the fold-changes for these genes induced by the PA14 wild-type compared with the PAO1 wild-type, and the fold-changes induced by PA14 Δ*flgK* and Δ*popB* mutants compared with their parental PA14 strain. At 3 h, the levels of *il-18* and *il-1*β cytokine genes induced by PA14 wild-type infection of hCF were ~3-fold higher than the levels induced by PAO1 wild-type (*P* < 0.05; Figure 2A). At 6 h, PA14 induced statistically significant ~4-fold higher levels of *tlr4* (*P* < 0.05) compared with PAO1, whereas for the other genes, the observed fold-increases induced by both strains were similar (*P* > 0.05). hCF infection with the PA14 Δ*flgK* and Δ*popB* mutants, showed that at 3 h, there were significantly lower (~13-fold) levels of *il-18* (*P* < 0.001) and *il-1*β (*P* < 0.01) cytokine gene induction, compared with the PA14 wild-type strain (Figure 2A). By 6 h, infection with the PA14 Δ*flgK* mutant showed significantly decreased levels (~ 5–10-fold) of *tlr4, tlr5, nlrc4, casp-4, il-18*, and *il-1*β cytokine gene expression compared with PA14 wild-type (*P* < 0.05; Figure 2A). By contrast, infection with the PA14 Δ*popB* mutant showed significant reductions (~ 4-fold) in expression of *tlr4, tlr5, nlrc4*, and *il-18* genes (*P* < 0.05) compared to PA14 wild-type, but not significantly different for *casp-4* and *il-1*β genes (*P* > 0.05).

Protein levels detected by western blot were consistent with the levels of gene expression quantified by RT-qPCR. After 6 h of hCFs infection with wild-type PAO1 and PA14, significant levels of TLR4, TLR5, NLRC4, native form of caspase-4 and IL-1β and IL-18 mature-formed proteins were detected by western blot with quantification of protein densitometry ratios relative to endogenous β-actin (Figure 2B, Supplementary Table 2). Significantly lower levels of production of these molecules were induced by the PA14 Δ*flgK* and Δ*popB* mutant strains (*P* < 0.05) and the percentage reductions in inflammatory molecule protein densitometry values are shown in Supplementary Table 2. In comparison with wild-type bacteria, infection with the complemented PA14 Δ*flgK* pUCP19-*flgK* and PA14 Δ*popB* pUCP19-*popB* strains restored to varying degrees the protein levels of TLR4, TLR5, NLRC4, caspase-4 and mature IL-1β and IL-18 (Figure 2B, Supplementary Table 2). Thus, complementation of Δ*flgK* restored ~50, 85, 60, 40, 32, and 61% of TLR4, TLR5, NLRC4, Caspase-4, IL-18, and IL-1β protein levels, respectively (Supplementary Table 2). Complementation of Δ*popB* restored ~65, 60, 62, 57 37, and 73% of TLR4, TLR5, NLRC4, Caspase-4, IL-18, and IL-1β protein levels, respectively (Supplementary Table 2).

Protein levels of the caspase-4 pro-enzyme, and IL-18 and IL-1β pro-forms were also analyzed by western blot. Caspase-4 pro-enzyme and pro-IL-18 protein expression was found to be constitutive, whereas pro-IL-1β was induced by *P. aeruginosa* wild-type infection, reduced by infection with the Δ*popB* mutant and very weakly induced by infection with the PA14 Δ*flgK* mutant (Figure 2C).

### hCFs infected with *P. aeruginosa* release extracellular IL-1β but not IL-18

**Figure 3:**
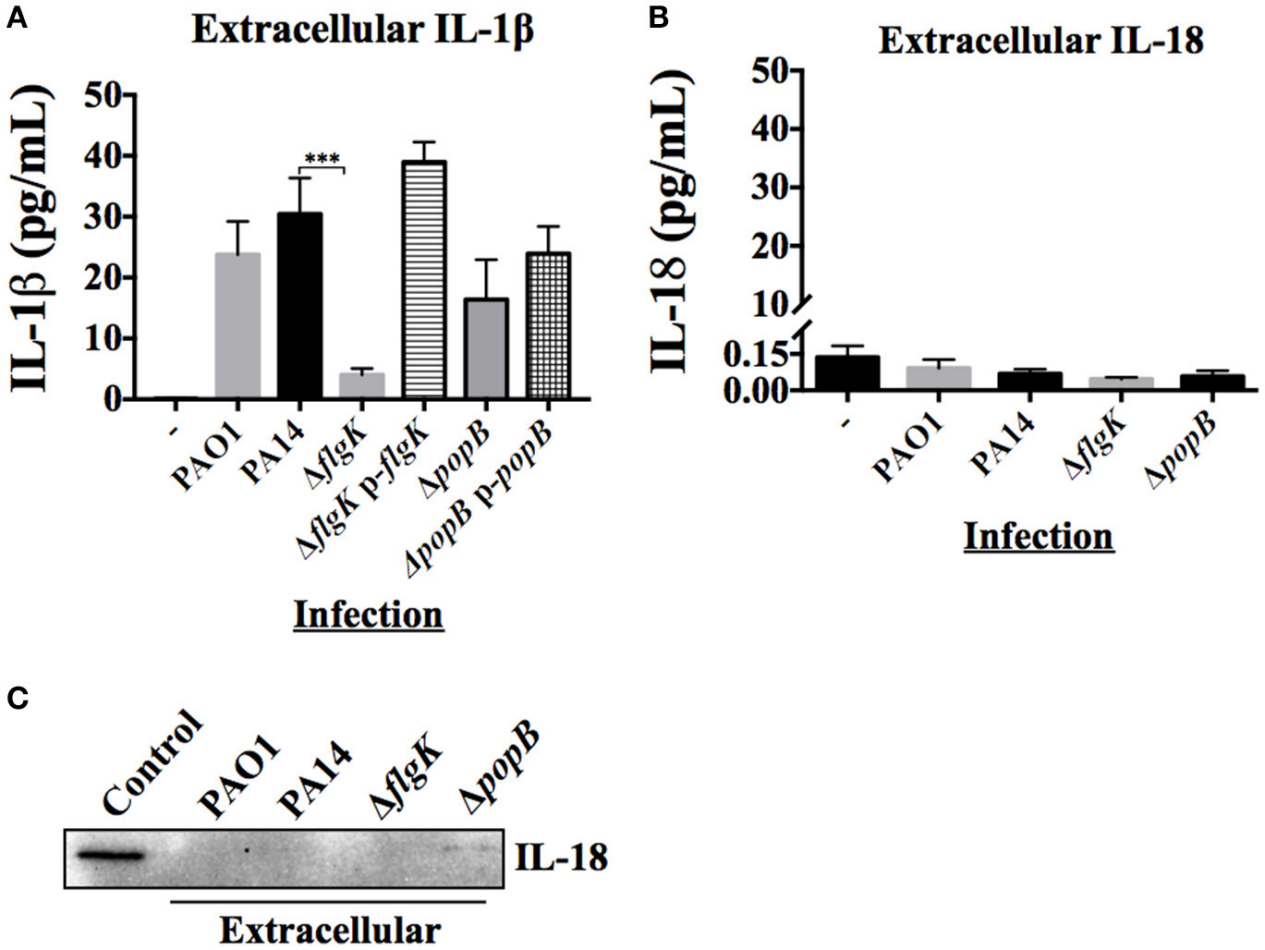
**Extracellular levels of IL-18 and IL-1β cytokines during hCFs infection with *P. aeruginosa***. IL-1β and IL-18 cytokine release was measured after 6 h hCFs infection with PAO1 and PA14 wild-type and PA14 Δ*flgK* and PA14 Δ*popB* mutant and complemented strains (MOI = 10). The columns represent the mean and error bars the SEM from *n* = 3 independent experiments. MSD-ELISA was used to quantify the levels of extracellular mature IL-1β **(A)** and IL-18 **(B)**. Statistical significance was calculated with a one-way ANOVA with Dunnett's multiple comparison test and it is represented by asterisks as follows: ^***^P < 0.001. **(C)** Extracellular IL-18 detected by western blot in TCA-precipitated supernatants after 6 h infection of hCFs with *P. aeruginosa* wild-type and mutant strains. A total protein extract from hCFs infected with PA14 was used as a positive control for IL-18 detection (Control).

The inflammatory cytokine profile of hCFs infected with wild-type *P. aeruginosa* is characterized predominantly by the production of IL-1β and IL-18 with the expression of other cytokines and chemokines not significant (manuscript submitted elsewhere). The early release of mature IL-1β and IL-18 was quantified by MSD-ELISA from the supernatants of hCFs infected with wild-type PAO1 and PA14 strains and isogenic PA14 Δ*flgK* and Δ*popB* mutants after 6 h. No significant differences in IL-1β were found between PAO1 and PA14 (*P* > 0.05). Both *P. aeruginosa* wild-type strains induced ~30 pg/mL of IL-1β secretion in hCFs after 6 h of infection (Figure 3A). By contrast, a significant reduction in IL-1β secretion to levels of ~5 pg/mL was observed from hCFs infected with the PA14 Δ*flgK* mutant strain. These lower levels of extracellular IL-1β detected in the supernatants of hCFs infected with the PA14 Δ*flgK* are in agreement with the low intracellular IL-1β levels shown in Figure 2B under the same infection conditions. There were no significant differences (*P* > 0.05) in the levels of IL-1β secretion induced by the PA14 Δ*popB* mutant (~20 pg/mL) compared to wild-type PA14 after 6 h (Figure 3A). Complementation of each mutation restored the levels of IL-1β release similar to those induced by the PA14 wild-type strain.

Measurement of IL-18 in infected hCFs supernatants revealed levels of extracellular IL-18 of ≤0.25 pg/mL for all infection conditions, which was similar to uninfected controls (*P* > 0.05; Figure 3B). Western blotting of IL-18 protein precipitated from infected hCF-supernatants collected after 6 h infection did not show the presence of mature IL-18 (Figure 3C), which was in agreement with the MSD-ELISA data.

### Signal pathway inhibition in hCFs appears to influence IL-1β and IL-18 expression and release

**Figure 4:**
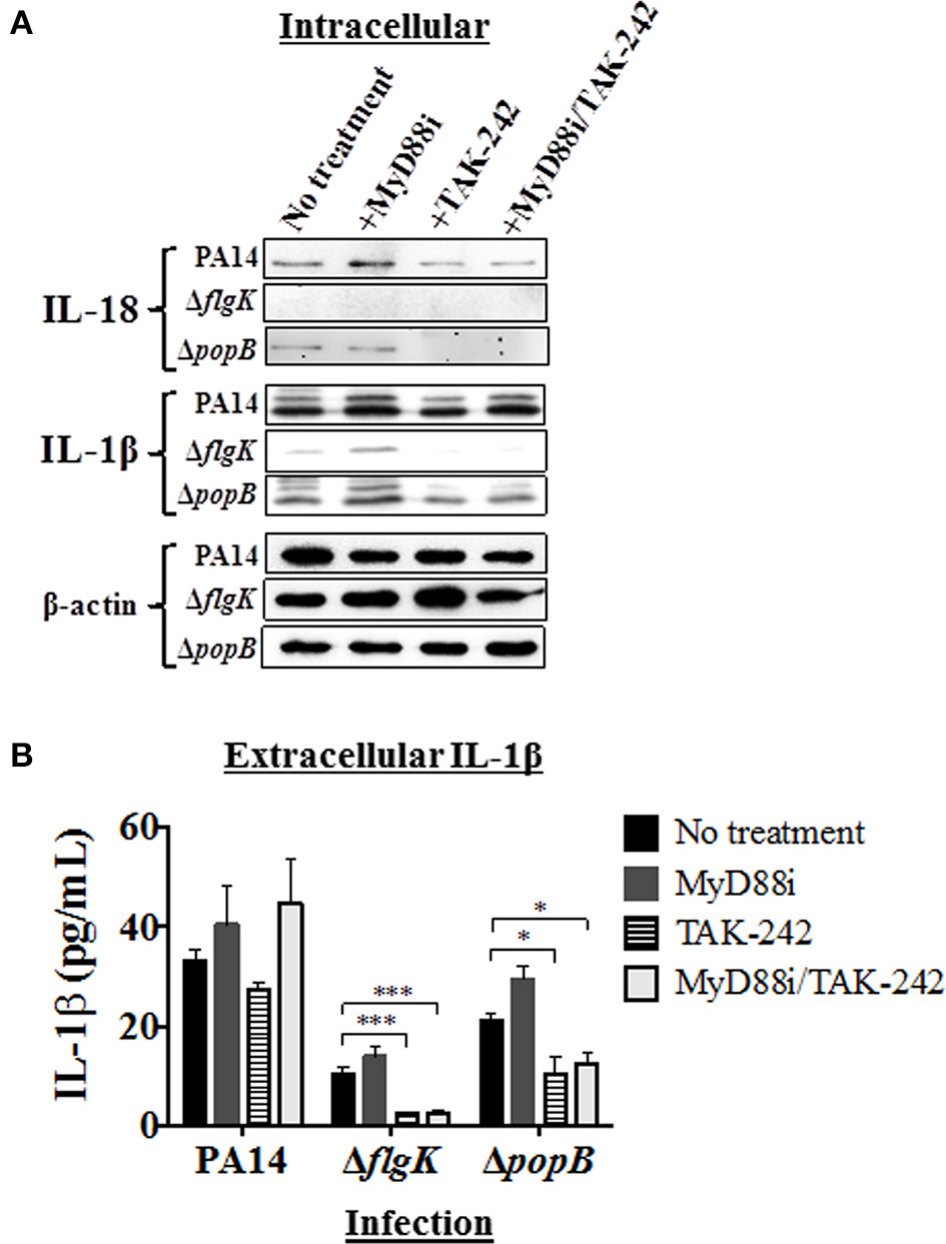
**Effects of pathway inhibition on IL-18 and IL-1β production**. Cells were treated with MyD88i, TAK-242, MyD88i/TAK-242 inhibitors and then infected with PAO1, PA14, Δ*flgK* and Δ*popB* strains (MOI = 10). **(A)** Western blot was used to detect intracellular mature IL-18 and IL-1β cytokines after 6 h of bacterial infection. Detection of intracellular levels of β-actin was used as endogenous and loading control. **(B)** Quantification of extracellular IL-1β cytokine after 6 h of infection. The columns represent the means and the error bars the SEM of *n* = 3 independent experiments. Statistical significance was calculated with a one-way ANOVA with Dunnett's multiple comparison test and it is represented by asterisks as follows: ^*^P < 0.05, ^***^P < 0.001.

Pathway inhibition was used to target key intracellular signaling molecules stimulated in hCFs by *P. aeruginosa* flagellum and TTSS that potentially could contribute to IL-18 and IL-1β production. Since our results showed similar cytokine production in hCFs caused by PAO1 and PA14 infection, only infection with PA14 was used for the subsequent inhibition experiments. Confluent monolayers were treated with MyD88i, TAK-242, and MyD88i/TAK-242 inhibitors and then infected with PA14 wild-type and PA14 Δ*flgK* and Δ*popB* mutant strains for 6 h. MyD88i blocks the signal through the MyD88 molecule and TAK-242 interferes with the association between the TIRAP and TRAM molecules to TLRs and inhibits the consequent signaling triggered by the receptor. Although TAK-242 has been reported to have selectivity for the LPS receptor TLR4, inhibitory effect has also been seen with other TLRs including TLR5 (Loiarro et al., 2005; Takashima et al., 2009; Matsunaga et al., 2011). Intracellular IL-18 and IL-1β protein expression was examined by western blot (Figure 4A) and extracellular release of these cytokines was quantified by MSD-ELISA (Figure 4B). Treatment of hCF monolayers with MyD88i, TAK-242 alone and MyD88i/TAK-242 together, did not induce IL-18 or IL-1β production (Supplementary Figure 3).

Infection with PA14 wild-type induced intracellular IL-18 protein production and protein densitometry values (from *n* = 3 independent western blots) revealed no significant differences in intracellular IL-18 protein expression induced by addition of any of the inhibitors (Figure 4A and Supplementary Table 3). Infection with the PA14 Δ*popB* mutant also induced mature IL-18 intracellular expression, but addition of TAK-242 (alone or in combination with MyD88i) appeared to significantly decrease its intracellular production (*P* < 0.01; Figure 4A and Supplementary Table 3), whereas MyD88i alone had no significant effect. PA14 Δ*flgK* did not induce intracellular IL-18 production, and secretory IL-18 was absent in all treated hCFs, as previously observed (Figures 3B,C).

Infection with wild-type PA14 induced intracellular IL-1β protein production, which was not inhibited by MyD88i or TAK-242 (Figure 4A and Supplementary Table 3). In cells infected with PA14 wild-type, levels of extracellular IL-1β were apparently stimulated by the addition of MyD88i or the MyD88i/TAK-242 combination, but no statistically significant differences were found compared to untreated cells (*P* > 0.05, Figure 4B). PA14 Δ*flgK* mutant induced low levels of intracellular and extracellular IL-1β in agreement with the results in Figures 2B, 3B, and were null with the addition of TAK-242, alone or in combination with MyD88i (Figures 4A,B and Supplementary Table 3B). Addition of TAK-242 also appeared to diminish intracellular and extracellular IL-1β production in hCFs infected with the PA14 Δ*popB* mutant (Figures 4A,B and Supplementary Table 3).

### TLR5 silencing in hCFs decreases IL-18 and IL-1β expression and release

Our results showed that infection with the PA14 Δ*flgK* mutant led to reduced IL-18 and IL-1β expression by hCFs. In order to demonstrate that the flagellum may be involved in inducing cytokine expression by these cells, we next tested the hypothesis that silencing the flagellum receptor TLR5 in hCFs would lead to a reduction of cytokine production regardless of bacterial adhesion. For these experiments, the flagellum filament protein FliC of *P. aeruginosa* was used as a control to demonstrate TLR5 stimulation. Recombinant (r) FliC was cloned and over-expressed in *E. coli* using the pET expression system (Supplementary Figure 1). Wild-type hCFs and hCFs that were TLR5-silenced using siRNA-TLR5 were stimulated with the PA14 wild-type, Δ*flgK* mutant strain and with 5 μg/mL of rFliC, and IL-18 and IL-1β gene expression was measured by RT-qPCR. Non-stimulated cells were used as a control.

**Figure 5:**
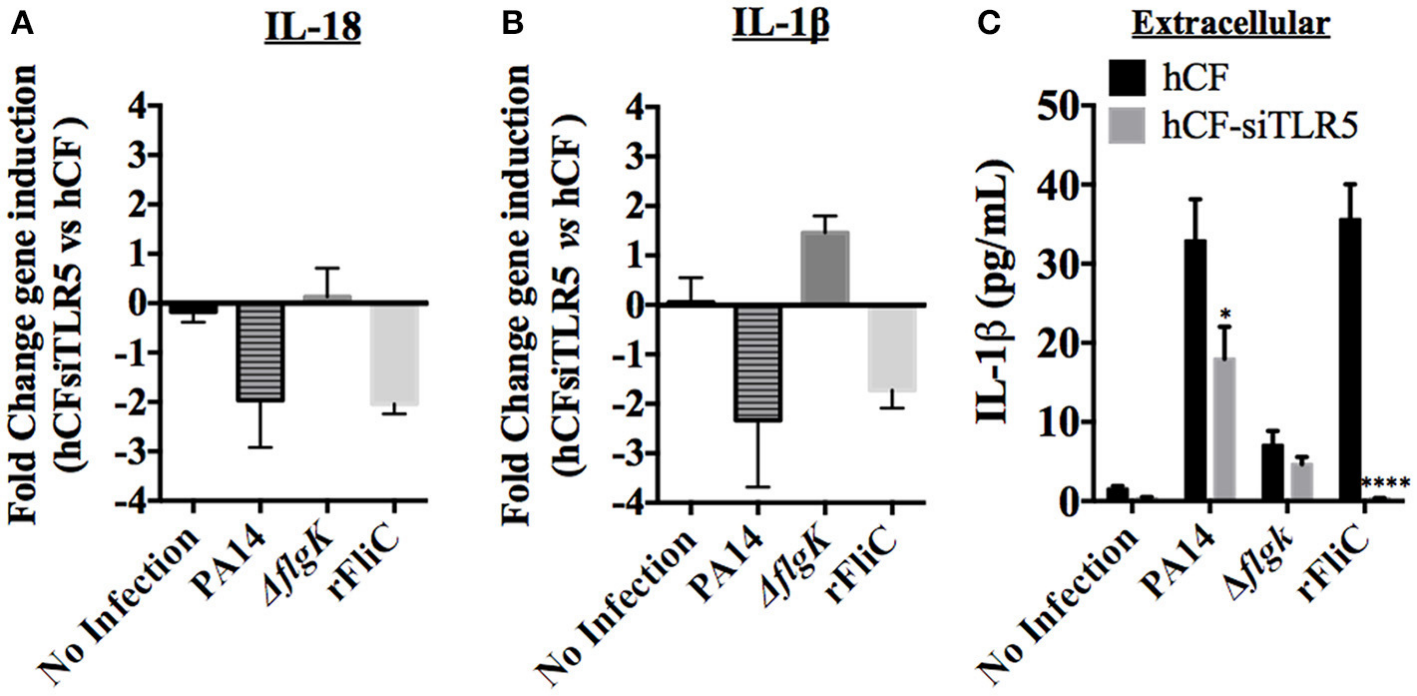
**Effect of TLR5 silencing in hCFs on IL-18 and IL-1β expression and release**. Wild-type hCFs and siTLR5-hCFs were infected with PA14 wild-type and Δ*flgK* mutant strain at MOI = 10, and stimulated with 5 μg/mL of rFliC for 6 h. LAL assay quantified that rFliC contained ~150 pg of LPS/mg of protein (<0.00002% w/w LPS) and RT-qPCR showed that these levels of *E. coli* LPS did not stimulate any gene transcription in hCFs (data not shown). Non-infected monolayers were used as a control. **(A,B)** Gene expression of IL-18 **(A)** and IL-1β **(B)** was measured by RT-qPCR. The bars show the fold changes in gene expression in siTLR5-hCFs compared to wild-type hCFs, which were determined using the 2^−ΔΔ*CT*^ method (Livak and Schmittgen, 2001). **(C)** Extracellular IL-1β levels measured by MSD-ELISA. rFliC was delivered intracellularly using SLO treatment. SLO treatment alone did not stimulate significant IL-1β release by hCFs (data not shown). The graph shows the levels of extracellular IL-1β quantified in the supernatants of hCFs and siTLR5-hCFs after 6 h following the different infection and stimulatory conditions. Statistical significance was calculated with a one-way ANOVA with Dunnett's multiple comparison test and it is represented by asterisks as follows: ^*^P < 0.05, ^****^P < 0.0001.

Control experiments demonstrated that hCFs transfected with siRNA-TLR5 and infected with PA14 for 6 h significantly diminished TLR5 gene (*P* < 0.0001; Supplementary Figure 4A) and protein (Supplementary Figure 4B) expression compared to control Mock transfection. Expression of *il-18* gene was reduced ~2 fold in hCF-siTLR5 compared to hCFs when stimulated with PA14 and rFliC for 6 h (Figure 5A). Infection with PA14 Δ*flgK* mutant did not induce IL-18 RNA expression and TLR5-silencing did not affect IL-18 expression, which is in agreement with the results shown in Figure 4A. Similarly, decreased levels of IL-1β RNA expression in hCFs-siTLR5 were found following stimulation with PA14 and rFliC for 6 h (Figure 5B). Otherwise, IL-1β RNA expression was slightly increased in hCFs-siTLR5 infected with the PA14 Δ*flgK* mutant compared to no infection (Figure 5B).

Secreted cytokines (IL-18 and IL-1β) were quantified from each supernatant after 6 h of hCF and hCF-siTLR5 incubation with PA14, Δ*flgK* mutant and rFliC (Figure 5C). SLO-treatment was used to deliver rFliC intracellularly. As expected, no extracellular IL-18 was detected. Levels of secreted IL-1β protein were significantly reduced in supernatants of hCFs-siTLR5 compared to those detected in hCFs, both after stimulation with PA14 (*P* < 0.05 and ~30% of reduction). Most significantly, hCFs-siTLR5 treated with rFliC showed a reduction of IL-1β secretion of >95% (*P* < 0.0001) compared to wild-type cells (Figure 5C), which was similar to the data obtained by RT-qPCR (Figure 5B). No significant extracellular IL-1β secretion was induced by SLO treatment alone of hCFs (~0.7 pg/mL, data not shown).

**Figure 6:**
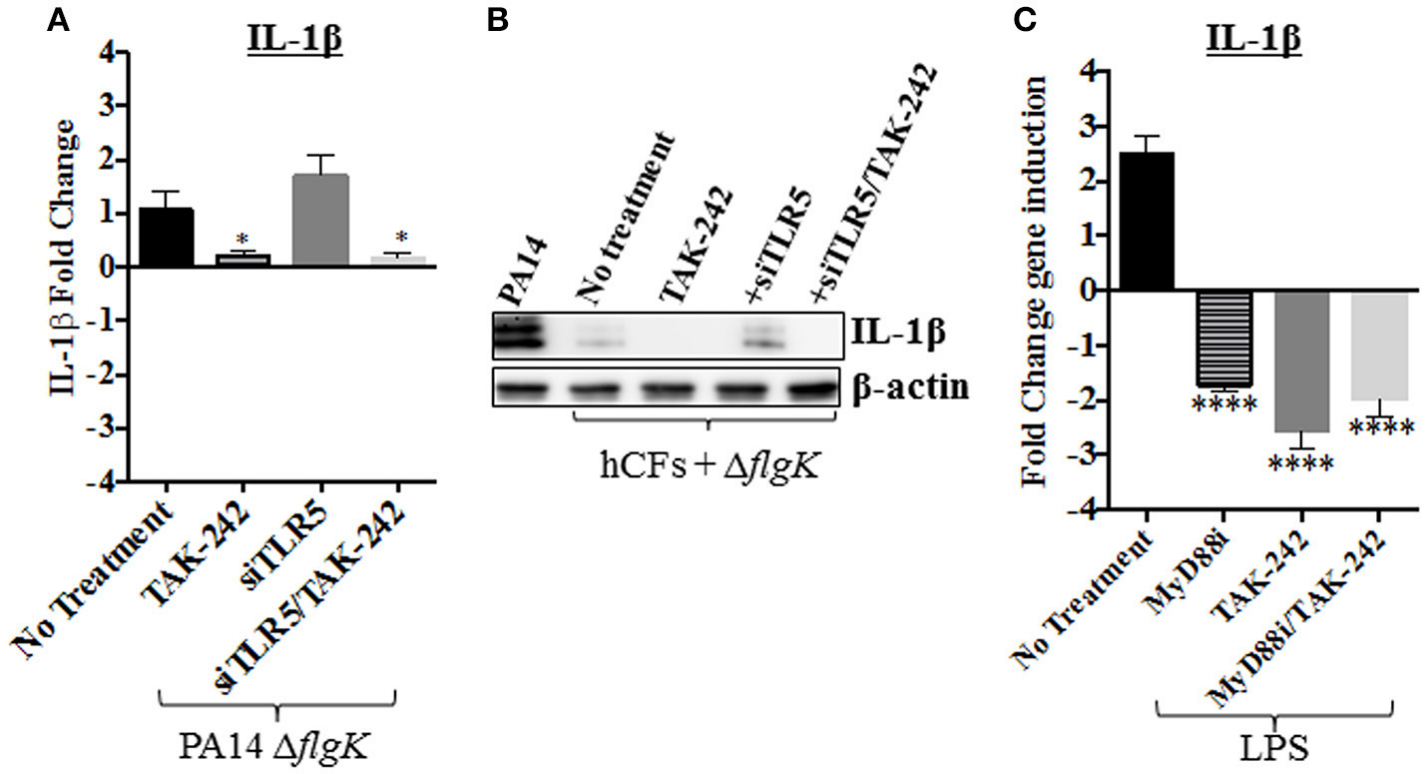
**Effect of PA14 Δ*flgK* mutant LPS on IL-1β expression by hCFs**. Wild-type hCFs and siTLR5-hCFs monolayers were treated with the TAK-242 inhibitor and then infected with PA14 Δ*flgK* mutant strain at MOI = 10 for 6 h. Un-treated and uninfected monolayers were used as control. **(A)** Gene expression of IL-1β was measured by RT-qPCR. The graph shows the fold changes of IL-1β expression calculated relative to the corresponding un-infected cell monolayer (hCF or siTLR5-hCF). **(B)** Intracellular IL-1β mature protein measured by western blot. A total protein extract from hCFs infected with PA14 was used as a positive control for IL-1β detection and intracellular levels of β-actin detected as an endogenous and loading control. **(C)** Gene expression of IL-1β in hCF monolayers un-treated and treated with TAK-242, Myd88i and TAK-242/Myd88i followed by stimulation with 100 ng/mL of *P. aeruginosa* LPS. The fold changes of IL-1β expression are calculated relative to un-stimulated monolayers. Statistical significance was calculated with a one-way ANOVA with Dunnett's multiple comparison test and it is represented by asterisks as follows: ^*^P < 0.05, ^****^P < 0.0001.

To examine if LPS was responsible, possibly, for the IL-1β induction observed in hCFs-siTLR5 infected with the PA14 Δ*flgK* mutant strain (Figure 5B), IL-1β gene expression was measured in hCFs-siTLR5 treated with the TAK-242 inhibitor. Under these inhibitory conditions, signaling through TLR5 (flagellin receptor) and TLR4 (LPS receptor) should be blocked. Repression of IL-1β expression was detected in both hCFs and hCFs-siTLR5 infected with PA14 Δ*flgK* mutant (*P* < 0.05, Figure 6A) treated with TAK-242. These transcriptional results were confirmed by detecting IL-1β protein expression under the same inhibitory conditions: addition of TAK-242 blocked the little IL-1β production induced in hCFs infected with the PA14 Δ*flgK* mutant (Figure 6B). In order to examine inhibitory effects on TLR4 sensing of LPS, IL-1β gene expression was also measured by RT-qPCR in hCFs that were stimulated with 100 ng/mL of pure *Pseudomonas* LPS, with and without TAK-242, Myd88i and TAK-242/Myd88i treatments. As shown in Figure 6C, addition of either inhibitor decreased IL-1β expression in hCFs treated with pure *Pseudomonas* LPS, compared to untreated cells. Similar experiments were done measuring IL-18 expression, but no increases in IL-18 expression were detected in LPS-stimulated hCFs (data not shown). No extracellular IL-1β protein was detected after hCF stimulation with *P. aeruginosa* LPS alone or in the presence of any inhibitor (data not shown).

### Treatment of hCF stimulated with *P. aeruginosa* rFliC with MyD88i and TAK-242 inhibits IL-18 and IL-1β expression

**Figure 7:**
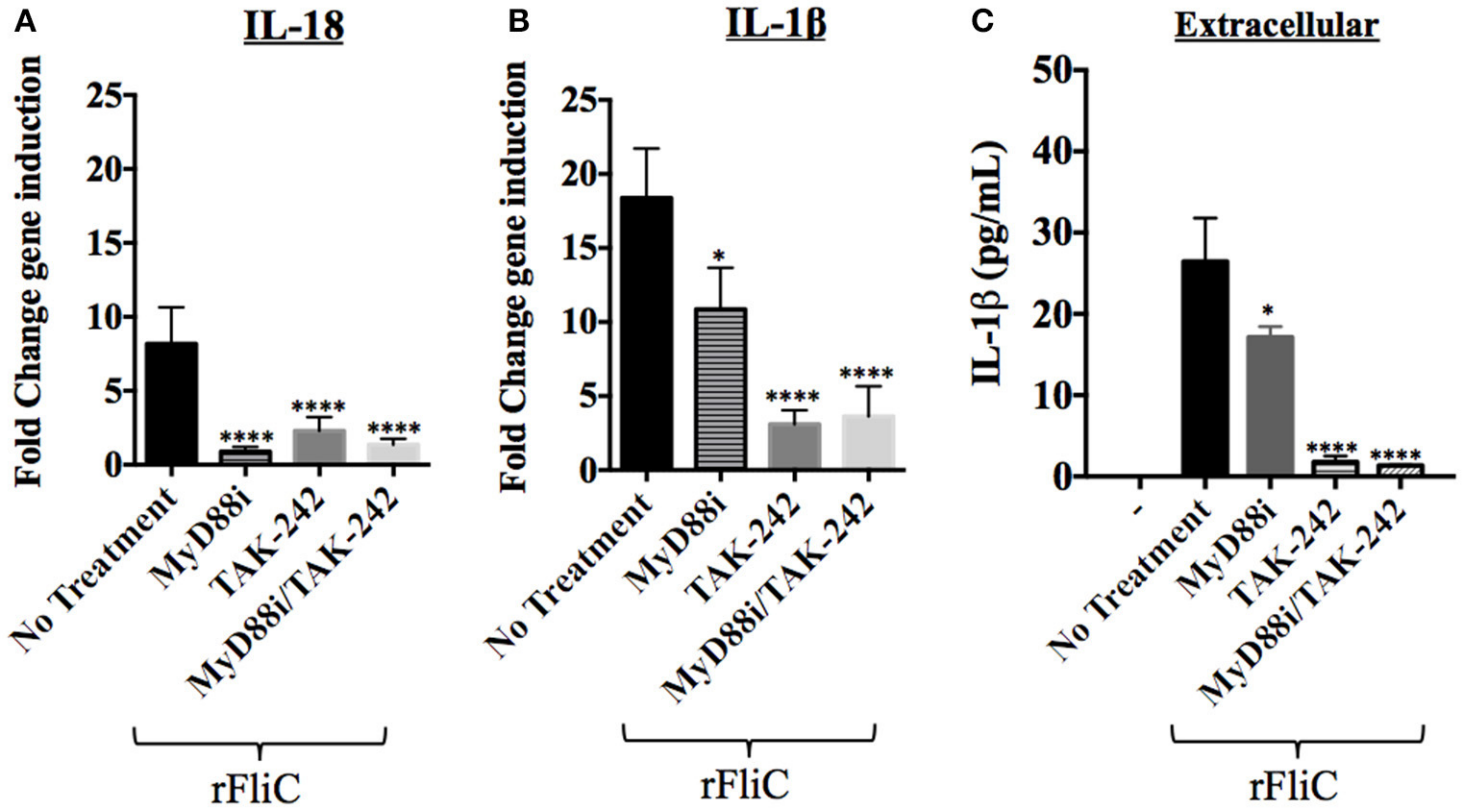
**Effect of MyD88i, TAK-242 and MyD88i/TAK-242 inhibition on IL-18 and IL-1β expression in hCF stimulated with rFliC**. IL-18 and IL-1β gene expression was measured by RT-qPCR from rFliC-stimulated hCF following treatment with MyD88i, TAK-242 and MyD88i/TAK-242 and untreated cells. The graph shows the fold changes of IL-18 **(A)** and IL-1β **(B)** gene expression calculated relative to the unstimulated and untreated hCF monolayers. The significance of the changes in gene expression shown in each inhibitory condition is compared to that measured in uninhibited hCF stimulated with *P. aeruginosa* rFliC. **(C)** Extracellular levels of IL-1β quantified from supernatants of inhibited hCFs following stimulation with rFliC. SLO-treatment was used to deliver rFliC intracellularly and no significant extracellular IL-1β was detected by treatment with SLO alone (data not shown). Unstimulated and inhibited cells were used as controls (-). Statistical significance was calculated with a one-way ANOVA with Dunnett's multiple comparison test and it is represented by asterisks as follows: ^*^P < 0.05, ^****^P < 0.0001.

Thus far, our data suggest that the *P. aeruginosa* flagellin (FliC) appears to plays an important role in inducing IL-18 and IL-1β expression through TLR5 stimulation. We tested the hypothesis that chemical inhibition of TLR5-mediated signaling pathways reduces cytokine gene expression induced by FliC. hCF monolayers were pre-treated with MyD88i, TAK-242 and both inhibitors combined, then stimulated with rFliC for 6 h and cytokine gene expression quantified by RT-qPCR (Figure 7). Controls were hCF monolayers without chemical pre-treatment and no stimulation with rFliC.

rFliC stimulation of hCF increased IL-18 gene expression ~10 fold compared to unstimulated cells and addition of MyD88i, TAK-242 and MyD88i/TAK-242 significantly reduced gene expression (*P* < 0.0001, Figure 7A). Similarly, rFliC stimulation increased IL-1β expression by ~20 fold, compared to unstimulated monolayers (Figure 7B). Inhibition with MyD88i alone reduced IL-1β gene expression by ~40% (*P* < 0.05), whereas treatment with TAK-242 and MyD88i/TAK-242 combined decreased IL-1β gene expression by >75% (*P* < 0.0001) compared to untreated cells (Figure 7B). Secreted IL-1β was quantified from supernatants of hCF stimulated with rFliC alone or in presence of the inhibitors. SLO-treatment was used to deliver the purified protein intracellularly. As shown in Figure 7C, MyD88i treatment reduced release of IL-1β by ~20%. By contrast, addition of TAK-242, alone or in combination with MyD88i, significantly reduced extracellular IL-1β release by >95% (*P* < 0.0001) compared to non-inhibited rFliC-stimulated hCFs.

## Discussion

The major finding from our study was that flagellin-mediated TLR5 stimulation induces IL-1β and IL-18 expression in an *ex vivo* human model of *P. aeruginosa* keratitis using primary corneal fibroblasts (hCF), but that only IL-1β is released extracellularly. By contrast, the *P. aeruginosa* TTSS (PopB) appears to have a minor role for inducing these cytokines by hCFs. Bacterial activation of PPR and signaling molecules that lead to inflammasome activation are widely reported to induce IL-1β and IL-18 cytokines and to promote pyroptotic cell death (Lamkanfi and Dixit, 2012). In our study, expression of TLR4, TLR5, NLRC4, caspase-4, and matured IL-18 and IL-1β cytokines was induced in hCFs after infection with *P. aeruginosa* wild-type strains. We observed no up-regulation of TLR2 or NLRP3 in hCFs, which has also not been observed in mice infected with *P. aeruginosa* (Sutterwala et al., 2007; Sun et al., 2010). In addition, caspase-4, but not caspase-1, was detected in hCF cells infected with *P. aeruginosa*. Stimulation of caspase-4 in our human cell culture model agrees with previous studies done in mice, for which caspase-11, the murine caspase-4-homolog, was reported to be targeted by NLRC4 (Kayagaki et al., 2011). Furthermore, caspase-4 activation in human macrophages has also been detected after infection with the different Gram negative bacteria *Legionella pneumophila, Yersinia pseudotuberculosis* and *Salmonella enterica* serovar Typhimurium (Casson et al., 2015).

We observed that deletion of the flagellum (Δ*flgK*) resulted in lower adhesion of *P. aeruginosa* to hCFs, which is likely to be the main explanation for the reduced expression and activation of inflammatory-associated molecules as a consequence of lower PRR stimulation and intracellular delivery of FliC monomer. This mutant is capable of producing similar amounts of FliC as the wild-type strain, but cannot enable polymerization of FliC to produce a functional flagellum (Patankar et al., 2013). Intracellular FliC could possibly be delivered by the *P. aeruginosa* TTSS, which has been described already for J77A.1 macrophages infected with *P. aeruginosa* (Ince et al., 2015). It is possible that FliC could then bind human NAIP prior to NLRC4 stimulation, which has been reported similarly for human primary monocyte-derived macrophages infected with *S. enterica* serovar Typhimurium (Ince et al., 2015). In our study, the effects of deleting the TTSS PopB protein, despite showing some reduction in expression of inflammatory-associated molecules, appeared to have no effect on cytokine protein expression or release by hCFs. Therefore, our data suggest that FliC, rather than PopB, appears to play a more significant role in stimulating IL-1β and IL-18 production by hCF. Significantly, both cytokines were expressed intracellularly, but only IL-1β was released. It is possible that IL-18 Binding Protein (IL-18BP), which is constitutively expressed and binds IL-18 with high affinity and inhibits cytokine function (Dinarello et al., 2013), binds the mature IL-18 protein within hCFs and prevents cytokine release during *P. aeruginosa* infection in our model.

Both IL-1β and IL-18 expression by hCFs relied on TLR5 stimulation by *P. aeruginosa* flagellin. The inflammatory response triggered by the stimulation of TLR5 by *P. aeruginosa* flagellin has also been reported in human corneal epithelial cells (Zhang et al., 2003). However, stimulation through TLR4 by pure *Pseudomonas* LPS and LPS present on live bacteria, also appeared to contribute to inducing IL-1β, but not IL-18, expression in our model. These results confirm our observations of up-regulated TLR4 expression in hCFs stimulated with pure *P. aeruginosa* LPS (Wong et al., 2011), but this previous study only measured TLR4 induction after 24 h. In the early stages of infection in our model, it is possible that flagellin and LPS have dual roles in PRR activation and IL-1β production. Our data from experiments in which siTLR5-hCF knockout cells, infected with non-flagellated *Pseudomonas*, showed a minor induction of IL-1β expression, demonstrate that LPS probably contributes to cytokine induction. It has been reported that LPS stimulates the NLRP3 inflammasome (Guo et al., 2015). However, in our study, we found no expression of NLRP3 in hCFs infected with *Pseudomonas*: therefore, this may explain why we did not observe extracellular matured IL-1β production in our experiments with pure LPS. The dominant inducer appears to be the flagellin, and this conclusion is supported by studies of murine keratitis provoked by *P. aeruginosa* infection, wherein LPS stimulation of TLR4 occurred only in animals infected with a flagellin-mutant strain of *P. aeruginosa* (Sun et al., 2010).

In our study, inhibition of the signaling molecule MyD88 using MyD88i appeared to affect predominantly IL-18 expression in hCFs, rather than IL-1β. However, addition of TAK-242 significantly reduced and, in some cases, blocked hCF production of both cytokines. Activation through TLR5 is able to stimulate TRAM/TRIF molecules in addition to MyD88, e.g., as observed in human colonic epithelial cells and in murine corneal macrophages infected with *P. aeruginosa* (Choi et al., 2010; Sun et al., 2010). Since the inhibitory effects of TAK-242 have been also reported to occur through other TLRs (Matsunaga et al., 2011), we hypothesized that in hCFs, TLR5 could trigger signaling via stimulation of MyD88 and the TRAM-TRIF molecules for both IL-18 and IL-1β expression, with the former more dependent on signaling mediated through MyD88. However, detection of IL-18 and IL-1β expression in wild-type *P. aeruginosa-*infected hCF cultures that had been treated with Myd88i and TAK-242, suggests an alternative pathway for cytokine expression. In this model, IL-18 expression would be still dependent on flagellin sensing. Moreover, it is possible that this alternative mechanism could be influenced by TTSS-sensing, since only hCF infected with the PA14 Δ*popB* mutant inhibited with TAK-242, showed reduced IL-1β and null IL-18 protein expression. The involvement of the respective TTSS of *Yersinia pseudotuberculosis* and *S. typhimurium* in a mechanism of NF-κβ activation in a TLR-independent manner for cytokine production has been described in murine models and also in human embryonic kidney 293T cells *in vitro* (Hapfelmeier et al., 2005; Auerbuch et al., 2009).

In our study, we have shown that *P. aeruginosa* adheres to hCFs *in vitro*. Bacterial adhesion appears to be a key step for enabling PAMPs stimulation of cellular PRRs. We have also observed during the time-course of infection, that *P. aeruginosa* can invade hCFs and reside within membrane-bound vacuoles (manuscript submitted elsewhere). It is known that both IL-18 and IL-1β require the action of the inflammasome to mature into their active forms. NLRC4 is reported to sense the intracellular rod components of *Pseudomonas* TTSS and FliC (after binding the hNAIP protein) (Zhao et al., 2011), both PAMPs examined in this study. Furthermore, TLR5 is a PPR receptor anchored within the mammalian cell membrane and it is likely that only specific bacterial PAMP adhesion stimulates the downstream signaling pathway. However, the possibility that the process of invasion and the presence of intracellular *Pseudomonas* bacteria contribute to inflammasome activation cannot be excluded entirely and potential mechanisms require further study.

Infectious *Pseudomonas* keratitis can lead to visual impairment as a consequence of the interactions of the bacterium with host tissue, the triggered host innate inflammatory response that leads to the characteristic influx of neutrophils, and the therapeutic drugs (antibiotics and corticosteroids) used to treat the infection (Taube et al., 2015). Activation of the innate inflammatory response and neutrophil influx are essential host defenses needed to control *Pseudomonas* infection, and it is possible that inhibition of these host responses may be detrimental in the early stages of infection (Rock et al., 2010). However, over-stimulation of host defenses can cause significant disease pathology (Rock and Kono, 2008). Clinically observed corneal tissue damage is likely a consequence of *Pseudomonas* virulence attributes and the strength of the innate inflammatory response, and is exacerbated significantly by the activities of infiltrating neutrophils. Moreover, over-stimulation of the inflammatory response can continue even when the eye is sterilized with antibiotics (O'brien, 2003), with no alleviation of tissue damage. Although topical corticosteroids are frequently used in clinical treatment, this is a non-specific attempt to reduce inflammation and can be associated with ocular complications (Taube et al., 2015). On balance, dampening the gross inflammatory response in *Pseudomonas* keratitis is probably more beneficial than allowing pathogen clearance through natural host defenses that induce irreversible tissue damage and permanent sight loss. In the current study, our findings show how *P. aeruginosa* stimulates IL-18 and IL-1β expression in hCFs during the early stages of infection and they describe an important role for bacterial flagellin. We propose, therefore, that blocking flagellin-PRR-signaling interactions could be an adjunctive approach with antibiotic chemotherapy to ameliorate the initial inflammatory response toward *P. aeruginosa* infection in human keratitis.

## Author contributions

MdMC, PH, and MC designed the experimental research. MdMC performed the experiments. MdMC, PH, and MC interpreted and analyzed the data. MdMC, PH, and MC wrote the manuscript.

## Funding

MdMC, PH, and MC acknowledge the support from the Network for Antimicrobial Resistance and Infection Prevention (NAMRIP, University of Southampton).

### Conflict of interest statement

The authors declare that the research was conducted in the absence of any commercial or financial relationships that could be construed as a potential conflict of interest.

## Footnotes

^1^TIR: Toll/interleukin-1 receptor.

^2^MyD88: Myeloid differentiation primary response gene 88.

^3^TRIF: Toll/interleukin-1 receptor (TIR) domain containing adapter inducing interferon-β.

^4^TIRAP: Toll/interleukin-1 receptor (TIR) domain containing adapter protein.

^5^MAL: MyD88 adapter-like.

^6^TRAM: TRIF-related adaptor molecule.

^7^NF-κβ: Nuclear Factor Kappa-light-chain-enhancer of activated B cells.

^8^IRF: Interferon regulatory factor.

^9^NLRP: NLR family pyrin domain containing.

^10^AIM2: Absent in melanoma 2.

^11^NLRC: NLR family CARD domain containing.
