# Supplementary material for: Signaling Mediated by Toll-Like Receptor 5 Sensing of Pseudomonas aeruginosa Flagellin Influences IL-1β and IL-18 Production by Primary Fibroblasts Derived from the Human Cornea
Source: Front Cell Infect Microbiol. 2017 Apr 19;7:130. doi: 10.3389/fcimb.2017.00130 (PMC5395653; doi:10.3389/fcimb.2017.00130)
Supplement: Supplementary file 1 [file Table1.PDF]

Supplementary Table 1. Oligonucleotide primers used in this study.

Oligonucleotide	Sequence 5'⟶ 3'	Application
TLR4 up	CCCTGAGGCATTTAGGCAGCTA	RT-qPCR
TLR4 lw	AGGTAGAGAGGTGGCTTAGGCT	RT-PCR / RT-qPCR
TLR5 up	CAGAAACCTGCCCAACCTTAG	RT-qPCR
TLR5 lw	GATCCAAGCGAGTTAAAGCCTT	RT-PCR / RT-qPCR
NLRP3 up	TAGCCACGCTAATGATCGACT	RT-qPCR
NLRP3 lw	TTGATCGCAGCGAAGATCCAC	RT-PCR / RT-qPCR
NLRC4 up	GCAGCTGACCCTGAATGGC	RT-qPCR
NLRC4 lw	CTTGGTCAGAGCCTTGCAC	RT-PCR / RT-qPCR
Caspase-1 up	CGCTGGGACTCTCAGCAG	RT-qPCR
Caspase-1 lw	GATGTGGGCATAGCTGGG	RT-PCR / RT-qPCR
Caspase-4 up	ACGTATGGCAGGACAAATGC	RT-qPCR
Caspase-4 lw	GAGGGCATCTGTAGATTCTCCTG	RT-PCR / RT-qPCR
IL-1beta up	GAGGAAGATGCTGGTTCCCT	RT-qPCR
IL-1beta lw	AGGTGCATCGTGCACATAAC	RT-PCR / RT-qPCR
IL-18 up	CCTGGAATCAGATTACTTTGGC	RT-qPCR
IL-18 lw	CCGGGGTGCATTATCTCTAC	RT-PCR / RT-qPCR
β-actin up	CCTGGCACCCAGCACAAT	RT-qPCR
β-actin lw	GGGCCGGACTCGTCATAC	RT-PCR / RT-qPCR
PA14 flgK HindIII up	AAGCTTATGTCCGACCTACTCTCGATAG	Cloning
PA14 flgK BamHI lw	GGATCCTCAGCGGAACGCGCCTATCA	Cloning
PA14 popB HindIII up	AAGCTTATGAATCCGATAACGCTTGAACG	Cloning
PA14 popB BamHI lw	GGATCCTCAGATCGCTGCCGGTCG	Cloning
FliC NdeI up	AAACATATGGCCCTTACAGTCAACACGAAC	Cloning
FliC XhoI lw	AAACTCGAGGCGCAGCAGGCTCAGG	Cloning
T7 universal up	TAATACGACTCACTATAGGG	Clone Checking
T7 universal lw	GCTAGTTATTGCTCAGCGG	Clone Checking
M13 up	GTAAAACGACGGCCAGT	Clone Checking
M13 lw	CAGGAAACAGCTATCAC	Clone Checking

Supplementary Table 2. Protein densitometries of TLR4, TLR5, NLRC4, Caspase-4, IL-18 and IL-1β relative to β-actin. The tables compare the induction of TLR4, TLR5, NLRC4, Caspase-4, IL-18 and IL-1β in hCF infected with PAO1, PA14, PA14ΔflgK, PA14ΔpopB and complemented strains. Protein densitometries of the inflammatory molecules were calculated using the Quantity One 4.6.9 software (BioRad) and the calculations were done relative to the endogenous control β-actin. Raw data are shown for each molecule for the n=3 independent experiments with calculated means and standard deviations.* Reduction of protein levels after mutation of ΔflgK and ΔpopB in strain PA14 as a percentage (%) of the wild-type strain. **Restoration of protein levels after complementation of ΔflgK and ΔpopB in strain PA14 as a percentage (%) of the wild-type strain

**TLR4**	**Expt 1**	**Expt 2**	**Expt 3**	**Mean**	**Standard deviation**	**% reduction mutant protein levels***	**% restored complemented protein levels****
No infection	0.048	0.042	0.066	0.052	0.012		
PAO1	0.74	0.817	0.88	0.812	0.007		
PA14	1.052	0.979	1.108	1.046	0.065		
Δ*flgK*	0.048	0.045	0.057	0.050	0.006	93	
Δ*popB*	0.070	0.055	0.091	0.072	0.018	95	
Δ*flgK* pUCP19	0.040	0.031	0.055	0.042	0.012		
Δ*flgK*-pflgK	0.530	0.402	0.496	0.476	0.066		46
Δ*popB* pUCP19	0.381	0.299	0.490	0.390	0.096		
Δ*popB*-ppopB	0.622	0.601	0.817	0.680	0.119		65

**TLR5**	**Expt 1**	**Expt 2**	**Expt 3**	**Mean**	**Standard deviation**	**% reduction mutant protein levels***	**% restored complemented protein levels****
No infection	0.038	0.032	0.053	0.041	0.011		
PAO1	0.178	0.154	0.183	0.172	0.016		
PA14	0.378	0.306	0.459	0.381	0.077		

Δ*flgK*	0.023	0.019	0.054	0.032	0.019	92	
Δ*popB*	0.099	0.088	0.122	0.103	0.017	73	
Δ*flgK* pUCP19	0.074	0.062	0.107	0.081	0.023		
Δ*flgK*-pflgK	0.389	0.294	0.294	0.326	0.055		84
Δ*popB* pUCP19	0.012	0.010	0.009	0.010	0.002		
Δ*popB*-ppopB	0.281	0.153	0.256	0.230	0.068		60

**NLRC4**	**Expt 1**	**Expt 2**	**Expt 3**	**Mean**	**Standard deviation**	**% reduction mutant protein levels***	**% restored complemented protein levels****
No infection	0.052	0.050	0.072	0.058	0.012		
PAO1	1.140	0.753	1.050	0.981	0.203		
PA14	1.721	1.530	1.825	1.692	0.150		
Δ*flgK*	0.164	0.143	0.200	0.169	0.029	90	
Δ*popB*	0.365	0.299	0.295	0.320	0.039	81	
Δ*flgK* pUCP19	0.067	0.063	0.089	0.073	0.014		
Δ*flgK*-pflgK	1.005	1.013	1.009	1.009	0.004		60
Δ*popB* pUCP19	0.184	0.126	0.170	0.160	0.030		
Δ*popB*-ppopB	1.112	0.963	1.048	1.041	0.075		62

**CASPASE-4**	**Expt 1**	**Expt 2**	**Expt 3**	**Mean**	**Standard deviation**	**% reduction mutant protein levels***	**% restored complemented protein levels****
No infection	0.081	0.066	0.094	0.080	0.014		
PAO1	0.803	0.829	0.942	0.858	0.074		
PA14	1.100	0.994	1.302	1.132	0.156		
Δ*flgK*	0.085	0.059	0.067	0.070	0.013	94	
Δ*popB*	0.423	0.398	0.502	0.441	0.054	61	

Δ*flgK* pUCP19	0.075	0.041	0.095	0.070	0.028		
Δ*flgK*-pflgK	0.466	0.218	0.672	0.452	0.227		40
Δ*popB* pUCP19	0.355	0.325	0.421	0.367	0.049		
Δ*popB*-ppopB	0.872	0.386	0.692	0.650	0.246		57

**IL-18**	**Expt 1**	**Expt 2**	**Expt 3**	**Mean**	**Standard deviation**	**% reduction mutant protein levels***	**% restored complemented protein levels****
No infection	0.078	0.055	0.077	0.070	0.013		
PAO1	1.138	0.978	1.148	1.088	0.095		
PA14	1.101	1.199	1.223	1.174	0.065		
Δ*flgK*	0.032	0.016	0.043	0.030	0.013	97	
Δ*popB*	0.604	0.618	0.701	0.641	0.052	45	
Δ*flgK* pUCP19	0.056	0.040	0.053	0.050	0.008		
Δ*flgK*-pflgK	0.402	0.258	0.481	0.380	0.113		32
Δ*popB* pUCP19	0.051	0.045	0.060	0.052	0.007		
Δ*popB*-ppopB	0.417	0.343	0.559	0.440	0.110		37

**IL-1β**	**Expt 1**	**Expt 2**	**Expt 3**	**Mean**	**Standard deviation**	**% reduction mutant protein levels***	**% restored complemented protein levels****
No infection	0.051	0.037	0.063	0.050	0.013		
PAO1	1.021	0.917	1.233	1.057	0.161		
PA14	1.127	1.037	1.500	1.221	0.245		
Δ*flgK*	0.173	0.101	0.236	0.170	0.068	86	
Δ*popB*	0.794	0.716	0.833	0.781	0.059	36	
Δ*flgK* pUCP19	0.035	0.011	0.049	0.032	0.019		
Δ*flgK*-pflgK	0.710	0.678	0.862	0.750	0.098		61

Δ*popB* pUCP19	0.278	0.252	0.301	0.277	0.025	
Δ*popB*-ppopB	0.870	0.801	1.003	0.891	0.103	73

Supplementary Table 3. Cytokine densitometry ratios relative to the endogenous control β-actin. IL-18 and IL-1β protein ratios were calculated after western blot detection in hCF infected with PA14, PA14 Δ*flgK*, PA14 Δ*popB* after treatment with Myd88i, TAK-242, Myd88i/TAK-242. Raw data are shown from n=3 independent experiments, with calculated means and standard deviations.

**A) IL-18**	**NO TREATMENT**				
	**Expt 1**	**Expt 2**	**Expt 3**	**Mean**	**Standard deviation**
PA14	0.882	0.511	0.677	0.690	0.186
Δ*flgK*	0.001	0.000	0.000	0.000	0.001
Δ*popB*	0.120	0.079	0.131	0.110	0.027
	**MYD88i**				
	**Expt 1**	**Expt 2**	**Expt 3**	**Mean**	**Standard deviation**
PA14	0.740	0.910	0.810	0.820	0.085
Δ*flgK*	0.000	0.000	0.000	0.000	0.000
Δ*popB*	0.117	0.091	0.182	0.130	0.047
	**TAK-242**				
	**Expt 1**	**Expt 2**	**Expt 3**	**Mean**	**Standard deviation**
PA14	0.699	0.530	0.871	0.700	0.171
Δ*flgK*	0.000	0.000	0.000	0.000	0.000
Δ*popB*	0.000	0.010	0.020	0.010	0.010
	**MYD88i/TAK-242**				
	**Expt 1**	**Expt 2**	**Expt 3**	**Mean**	**Standard deviation**
PA14	0.640	0.810	0.680	0.710	0.089
Δ*flgK*	0.000	0.000	0.000	0.000	0.000
Δ*popB*	0.000	0.000	0.000	0.000	0.000

B) IL-1β	NO TREATMENT				
	Expt 1	Expt 2	Expt 3	Mean	Standard deviation
PA14	0.800	0.860	0.830	0.830	0.030
Δ*flgK*	0.075	0.071	0.094	0.080	0.012
Δ*popB*	0.401	0.391	0.348	0.380	0.028
	**MYD88i**				
	**Expt 1**	**Expt 2**	**Expt 3**	**Mean**	**Standard deviation**
PA14	0.910	1.107	0.953	0.990	0.104
Δ*flgK*	0.167	0.214	0.219	0.200	0.029
Δ*popB*	0.490	0.580	0.460	0.510	0.062
	**TAK-242**				
	**Expt 1**	**Expt 2**	**Expt 3**	**Mean**	**Standard deviation**
PA14	0.699	0.783	0.738	0.740	0.042
Δ*flgK*	0.001	0.000	0.000	0.000	0.001
Δ*popB*	0.178	0.120	0.152	0.150	0.029
	**MYD88i/TAK-242**				
	**Expt 1**	**Expt 2**	**Expt 3**	**Mean**	**Standard deviation**
PA14	0.713	0.689	0.908	0.770	0.120
Δ*flgK*	0.016	0.007	0.013	0.012	0.006
Δ*popB*	0.197	0.237	0.256	0.230	0.030
