# Supplementary material for: Signaling Mediated by Toll-Like Receptor 5 Sensing of Pseudomonas aeruginosa Flagellin Influences IL-1β and IL-18 Production by Primary Fibroblasts Derived from the Human Cornea
Source: Front Cell Infect Microbiol. 2017 Apr 19;7:130. doi: 10.3389/fcimb.2017.00130 (PMC5395653; doi:10.3389/fcimb.2017.00130)
Supplement: Supplementary file 2 [file Presentation1.PPTX]

## Slide 1
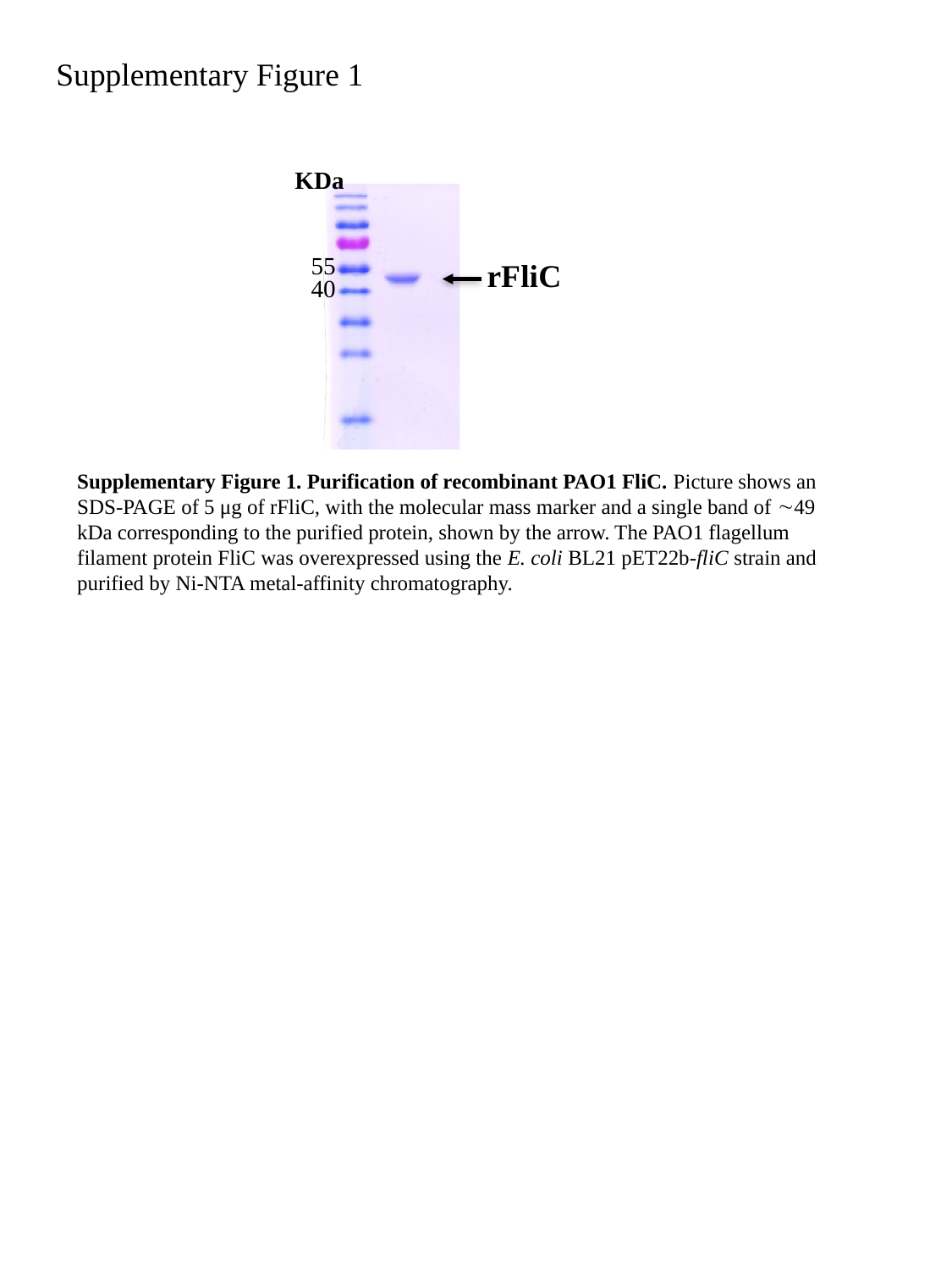

Supplementary Figure 1
KDa
55
rFliC
40
Supplementary Figure 1. Purification of recombinant PAO1 FliC. Picture shows an SDS-PAGE of 5 μg of rFliC, with the molecular mass marker and a single band of 49 kDa corresponding to the purified protein, shown by the arrow. The PAO1 flagellum filament protein FliC was overexpressed using the E. coli BL21 pET22b-fliC strain and purified by Ni-NTA metal-affinity chromatography.

## Slide 2
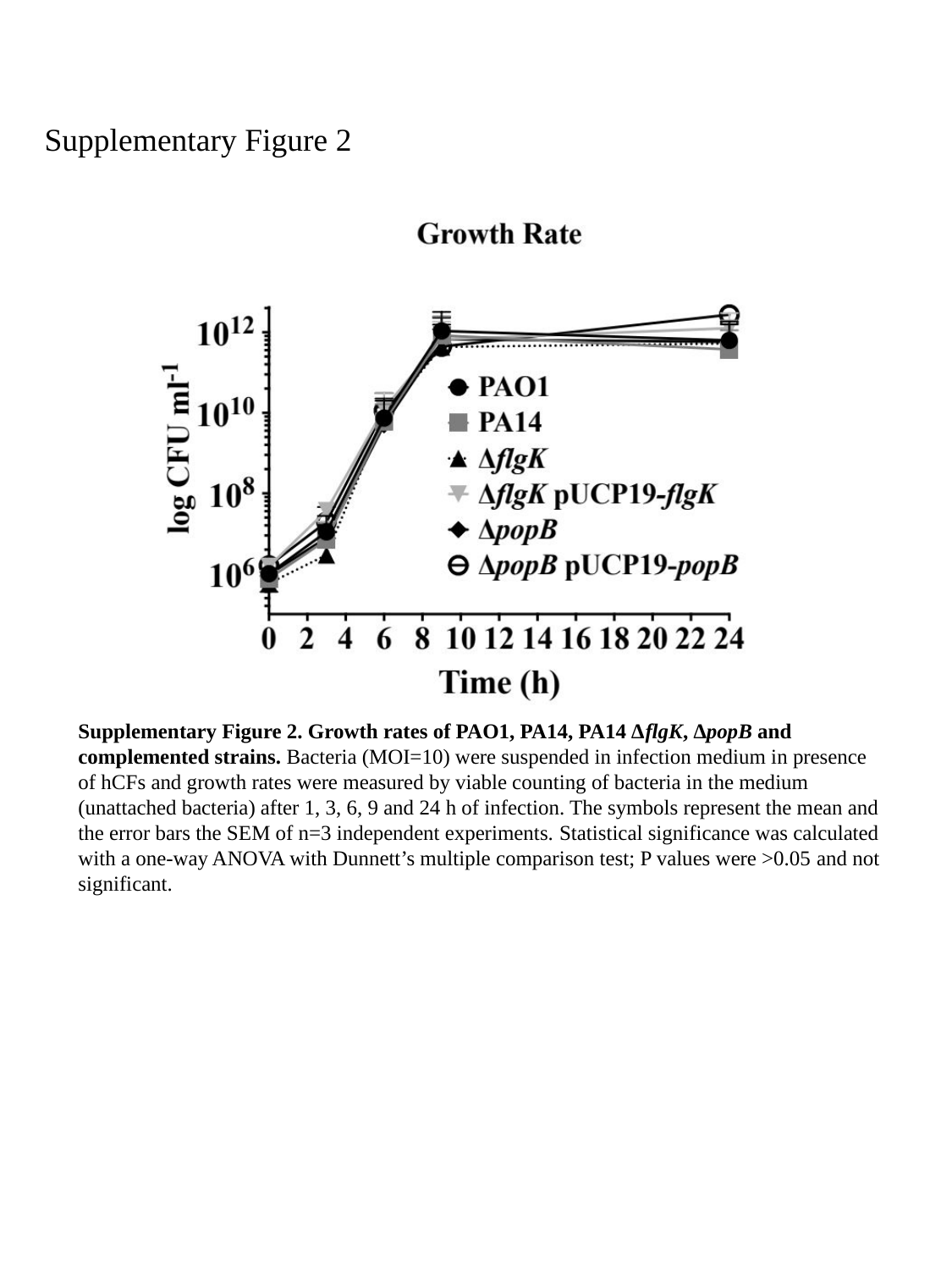

Supplementary Figure 2
Supplementary Figure 2. Growth rates of PAO1, PA14, PA14 ∆flgK, ∆popB and complemented strains. Bacteria (MOI=10) were suspended in infection medium in presence of hCFs and growth rates were measured by viable counting of bacteria in the medium (unattached bacteria) after 1, 3, 6, 9 and 24 h of infection. The symbols represent the mean and the error bars the SEM of n=3 independent experiments. Statistical significance was calculated with a one-way ANOVA with Dunnett’s multiple comparison test; P values were >0.05 and not significant.

## Slide 3
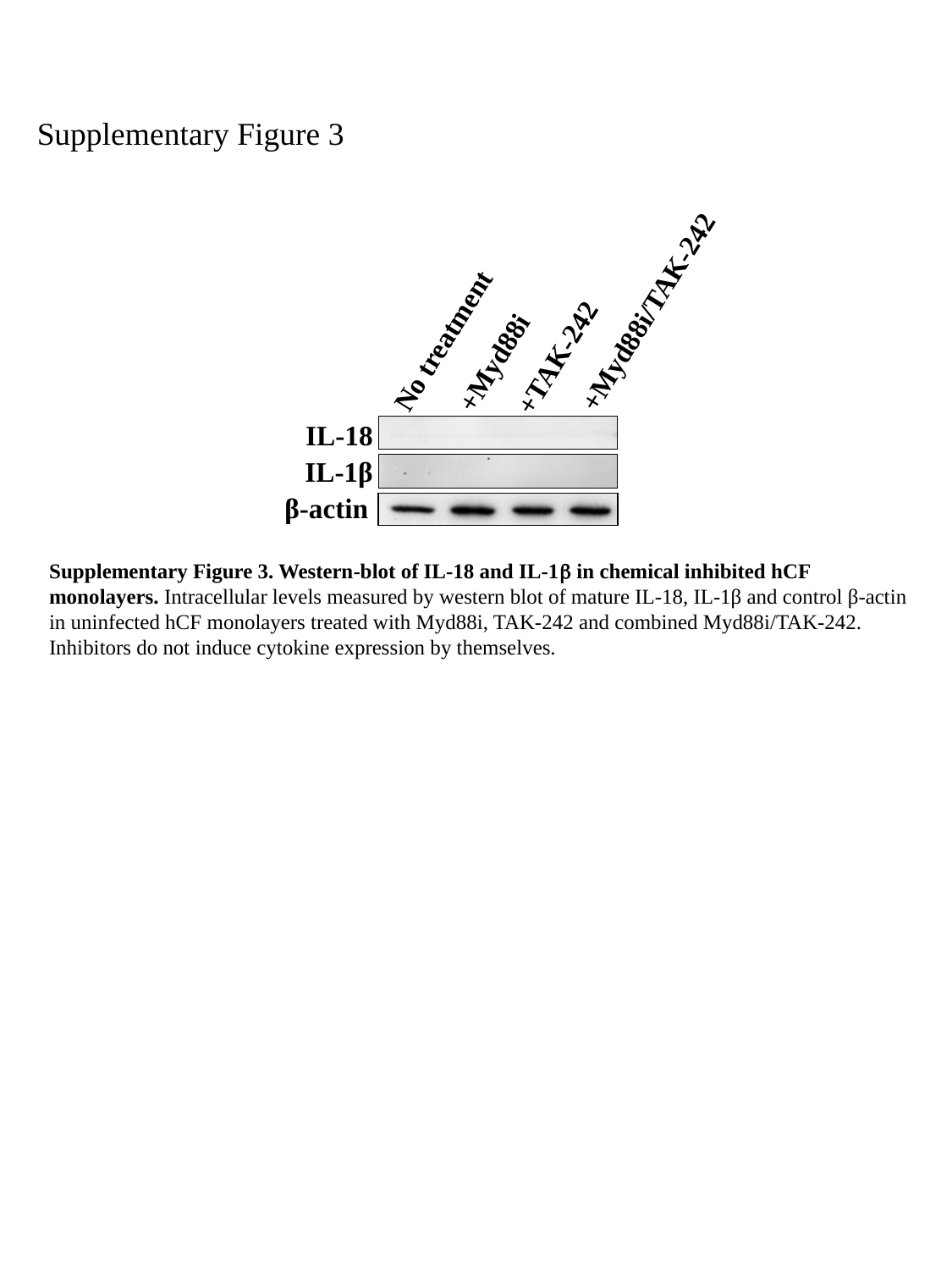

Supplementary Figure 3
+Myd88i/TAK-242
No treatment
+TAK-242
+Myd88i
IL-18
IL-1β
β-actin
Supplementary Figure 3. Western-blot of IL-18 and IL-1β in chemical inhibited hCF monolayers. Intracellular levels measured by western blot of mature IL-18, IL-1β and control β-actin in uninfected hCF monolayers treated with Myd88i, TAK-242 and combined Myd88i/TAK-242. Inhibitors do not induce cytokine expression by themselves.

## Slide 4
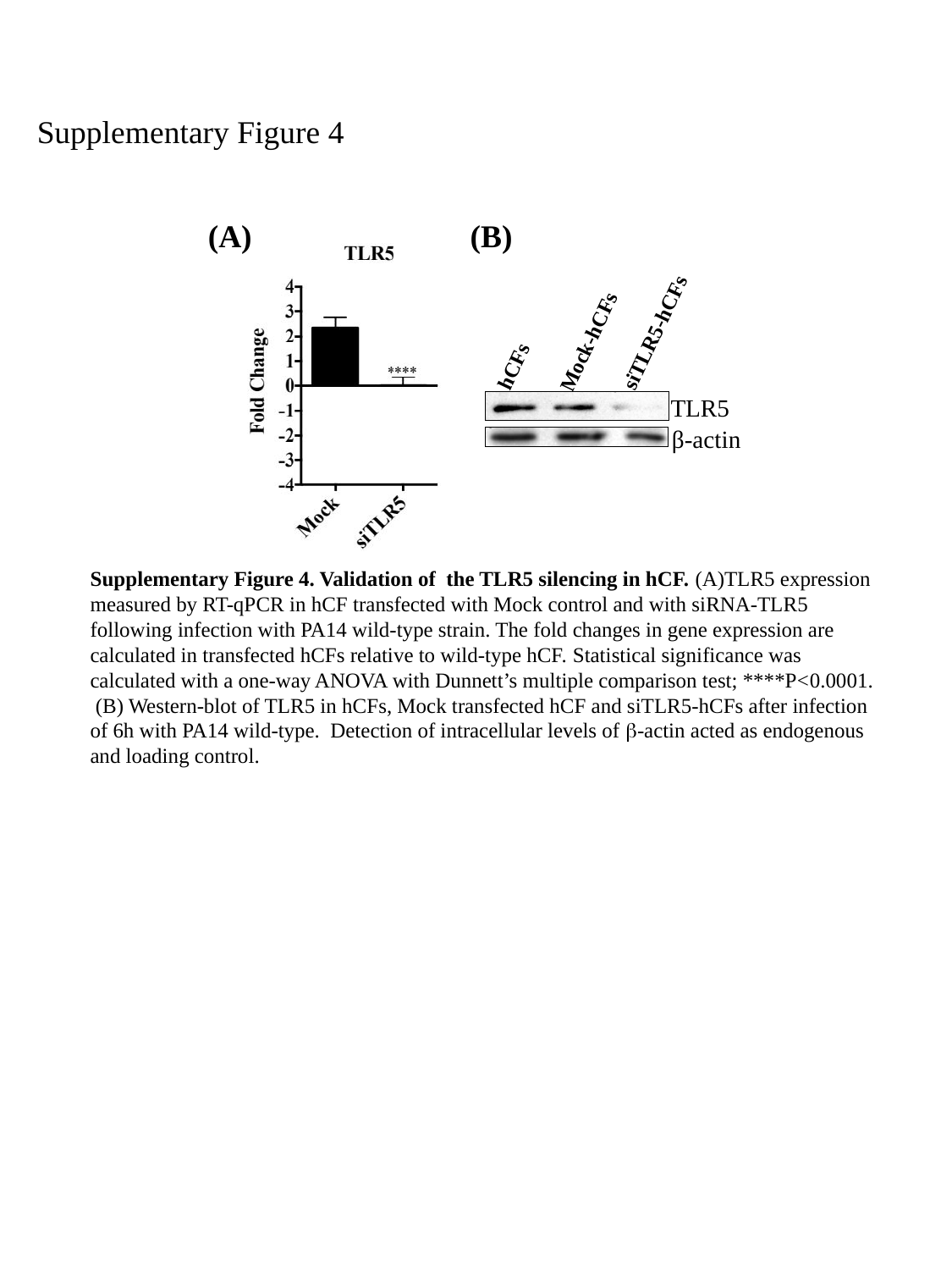

Supplementary Figure 4
(A)
(B)
siTLR5-hCFs
Mock-hCFs
hCFs
TLR5
β-actin
Supplementary Figure 4. Validation of the TLR5 silencing in hCF. (A)TLR5 expression measured by RT-qPCR in hCF transfected with Mock control and with siRNA-TLR5 following infection with PA14 wild-type strain. The fold changes in gene expression are calculated in transfected hCFs relative to wild-type hCF. Statistical significance was calculated with a one-way ANOVA with Dunnett’s multiple comparison test; ****P<0.0001.
 (B) Western-blot of TLR5 in hCFs, Mock transfected hCF and siTLR5-hCFs after infection of 6h with PA14 wild-type. Detection of intracellular levels of β-actin acted as endogenous and loading control.
